# Circular RNA vaccines with long-term lymph node-targeting delivery stability after lyophilization induce potent and persistent immune responses

**DOI:** 10.1128/mbio.01775-23

**Published:** 2023-12-11

**Authors:** Jiawu Wan, Zongmei Wang, Lingli Wang, Liqin Wu, Chengguang Zhang, Ming Zhou, Zhen F. Fu, Ling Zhao

**Affiliations:** 1 National Key Laboratory of Agricultural Microbiology, Huazhong Agricultural University, Wuhan, China; 2 Hubei Hongshan Laboratory, Wuhan, China; 3 Key Laboratory of Preventive Veterinary Medicine of Hubei Province, College of Veterinary Medicine, Huazhong Agricultural University, Wuhan, China; Virginia Polytechnic Institute and State University, Blacksburg, Virginia, USA

**Keywords:** CircRNA vaccine, lymph node-targeting delivery, lyophilization, humoral immunity, long-term protection

## Abstract

Several recent attempts to improve the stability and immunogenicity of messenger RNA (mRNA) vaccines include the use of circular RNA (circRNA), targeted delivery, and lyophilization. However, these research directions have often been pursued independently, ignoring the impact of the modification of lipid nanoparticle-encapsulated mRNA vaccines on their targeted delivery after lyophilization and on their subsequent immunogenicity. Here, we develop a circRNA vaccine targeting lymph nodes that expresses rabies virus glycoprotein (G), termed circRNA-G. Mannose modification is introduced directly into the process of synthesizing PEG lipids, and the resulting PEG-mannose can be used in the preparation of mannose-LNPs (mLNPs) that target dendritic cells, thereby promoting the specific distribution of circRNA-G to lymph nodes (mLNP-circRNA-G). We demonstrated that mLNP-circRNA-G has continuous antigen availability that promotes the generation of T follicular helper cells, germinal center B cells, long-lived plasma cells, and memory B cells in mice. Importantly, the vaccine with this targeting modification remained stable for at least 24 weeks of storage at 4℃ after lyophilization, and its immunogenicity was also maintained. Notably, this strategy also enhances the antibody production of the SARS-CoV-2 trimeric receptor-binding domain circRNA vaccine and the stability of immunogenicity after lyophilization. In summary, this study provides a general platform for the design of lyophilized vaccines with targeted stability, demonstrating the potential of lymph node-targeting circRNAs as next-generation vaccines.

**IMPORTANCE:**

messenger RNA (mRNA) vaccines are a key technology in combating existing and emerging infectious diseases. However, the inherent instability of mRNA and the nonspecificity of lipid nanoparticle-encapsulated (LNP) delivery systems result in the need for cold storage and a relatively short-duration immune response to mRNA vaccines. Herein, we develop a novel vaccine in the form of circRNAs encapsulated in LNPs, and the circular structure of the circRNAs enhances their stability. Lyophilization is considered the most effective method for the long-term preservation of RNA vaccines. However, this process may result in irreversible damage to the nanoparticles, particularly the potential disruption of targeting modifications on LNPs. During the selection of lymph node-targeting ligands, we found that LNPs modified with mannose maintained their physical properties almost unchanged after lyophilization. Additionally, the targeting specificity and immunogenicity remained unaffected. In contrast, even with the addition of cryoprotectants such as sucrose, the physical properties of LNPs were impaired, leading to an obvious decrease in immunogenicity. This may be attributed to the protective role of mannose on the surface of LNPs during lyophilization. Freshly prepared and lyophilized mLNP-circRNA vaccines elicited comparable immune responses in both the rabies virus model and the SARS-CoV-2 model. Our data demonstrated that mLNP-circRNA vaccines elicit robust immune responses while improving stability after lyophilization, with no compromise in tissue targeting specificity. Therefore, mannose-modified LNP-circRNA vaccines represent a promising vaccine design strategy.

## INTRODUCTION

The majority of licensed vaccines are thought to protect through the induction of antibody responses (1). Methods to enhance humoral immunity and particularly to promote the germinal center (GC) reaction where antibody affinity maturation occurs are of great interest for improved vaccines. For pathogens such as severe acute respiratory syndrome coronavirus 2 (SARS-CoV-2) and rabies virus (RABV), protein vaccines, inactivated vaccines, and messenger RNA (mRNA) vaccines can induce effective immune responses. However, the short duration of immunity resulting from antibody decline is an important limitation, particularly in the absence of enhanced immune processes (2). A potent vaccine leads to long-term—ideally lifelong—immunity against a specific pathogen, which can be achieved by eliciting both durable production from neutralizing antibodies (humoral immunity) and cellular immunity (3). Therefore, there is a critical need to develop protective vaccine strategies capable of inducing long-term antibody responses.

Despite the clear demonstration of efficacy for infectious disease and cancer vaccines, the application of mRNA is limited by the duration of expression, stability, and immunogenicity. mRNA exhibits a relatively short life cycle and is readily cleared by intracellular enzymes and the immune system, usually lasting only 1–3 days (4). Therefore, antigen expression of mRNA vaccines is relatively low, and repeated inoculation is required to obtain an adequate immune response. As an emerging generation of therapeutic mRNAs, circular RNAs (circRNAs) may address some of the limitations of linear mRNAs, offering increased stability and diminished autoimmunity (5, 6). Compared to linear mRNA, circRNA is highly stable due to its covalently closed ring structure, which protects it from exonuclease-mediated degradation (7). It has been reported that circRNAs have a median half-life at least 2.5 times longer than their linear mRNA isoforms in mammalian cells (8–10). With improved stability, the protein translation time of circRNA can last for nearly 1 week, and the protein yield of circRNA is increased hundreds of times compared with that of linear mRNA (2, 4, 5). Therefore, circRNA presents a better alternative for prolonged proteins expression. CircRNA not only provides greater antigenic stimulation but also distributes abundant expression of antigens for up to 1 week (4). This continued antigen translation allows the circRNA vaccine to function in a process similar to antigen slow release, which can more accurately mimic natural infection (11). This means that the circRNA vaccine can prolong vaccine exposure, which is necessary to boost the GC response (3).

Targeting vaccine components to specific immune cells in the lymph nodes (LNs) can improve vaccine responses. Efficient antigen presentation by antigen-presenting cells (APCs), such as dendritic cells (DCs), is a prerequisite for the high efficacy of mRNA vaccines (12, 13). The mannose receptor (MR), as an important pattern recognition and endocytosis receptor in the innate immune system, is present mainly on the cell membrane surface of dendritic cells and macrophages. DCs can ingest nanoparticles through MR-mediated endocytosis, so mannose-surface-modified nanoparticles preferentially bind to DCs. Modification with mannose endows the nanoparticles vaccine with good targeting ability *in vivo* and *in vitro* and with the ability to stimulate DC maturation (14). Nanoparticles that target DCs by using mannose-binding receptors elicit greater humoral and cellular immunity than nanoparticles alone (3). The uptake and retention of antigens in LNs can be increased by modifying nanoparticles to target LNs and, thus, improve GC formation, neutralizing antibody (nAb) concentration as well as memory B cell and long-lived plasma cell responses (15).

Existing mRNA vaccines heavily depend on lipid nanoparticle (LNP) delivery systems to improve their transfection efficiency *in vivo*. Although mRNA vaccines have advantages, challenges in physical and chemical stability still hinder their application (16). Although most conventional vaccines can be stored in a refrigerator between 2°C and 8°C for at least 6 months, lipid nanoparticle-encapsulated mRNA (LNP-mRNA) vaccines require cryopreservation. At present, lyophilized LNP-mRNA vaccines can be stored at room temperature for a long time, which has been achieved by an increasing number of laboratories (16–20). The process of lyophilization and rehydration introduces mechanical stress that can lead to damage to the liposome membrane (17). Although the lyophilization stability of LNPs can be improved by optimizing the lyophilization process and adding cryoprotectants, whether lyophilization damages the targeting modification of LNP surfaces has not been widely studied. At present, organ-specific mRNA vaccines and lyophilized mRNA vaccines are both promising strategies for developing next-generation mRNA vaccines. Therefore, designing a targeting modification strategy that can maintain targeted stability in lyophilization is key.

Here, we present a vaccine design strategy for the LN-targeting delivery of circRNA-induced durable immune responses, with nanoparticles featuring tunable drug loading, narrow size distribution, and high stability. Compared with untargeted modified LNP-circRNA-G, mannose-LNP-circRNA-G (mLNP-circRNA-G) improved nanoparticle retention, draining LN (dLN) accumulation, and cellular uptake of DCs, leading to persistent activation of DCs and B cells in dLNs and to significantly enhanced GC and antibody responses. We further demonstrate the importance of sustained antigenic stimulation and targeted LNs to induce a high-level and durable antibody response to RABV. The specific IgG and nAb were maintained at a high level for at least 6 months. A single dose of mLNP-circRNA-G protected the mice from RABV infection. Moreover, delivery of the SARS-CoV-2 trimeric receptor-binding domain (RBD) circRNA vaccine using targeting modification of LNPs also elicited robust antibody responses. Notably, by modifying mannose on PEG lipids and then mixing it with other excipients, mLNPs synthesized in one step remained stable in tissue targeting and robust immunogenicity after lyophilization. Collectively, our data in the mouse model suggested that the targeted delivery platform had promising performance in the design and generation of circRNA vaccines against RABV and SARS-CoV-2, which can potentially be expanded to vaccine development for other pathogens.

## RESULTS

### Design and characterization of mLNP-circRNA-G targeting DCs

**Fig 1:**
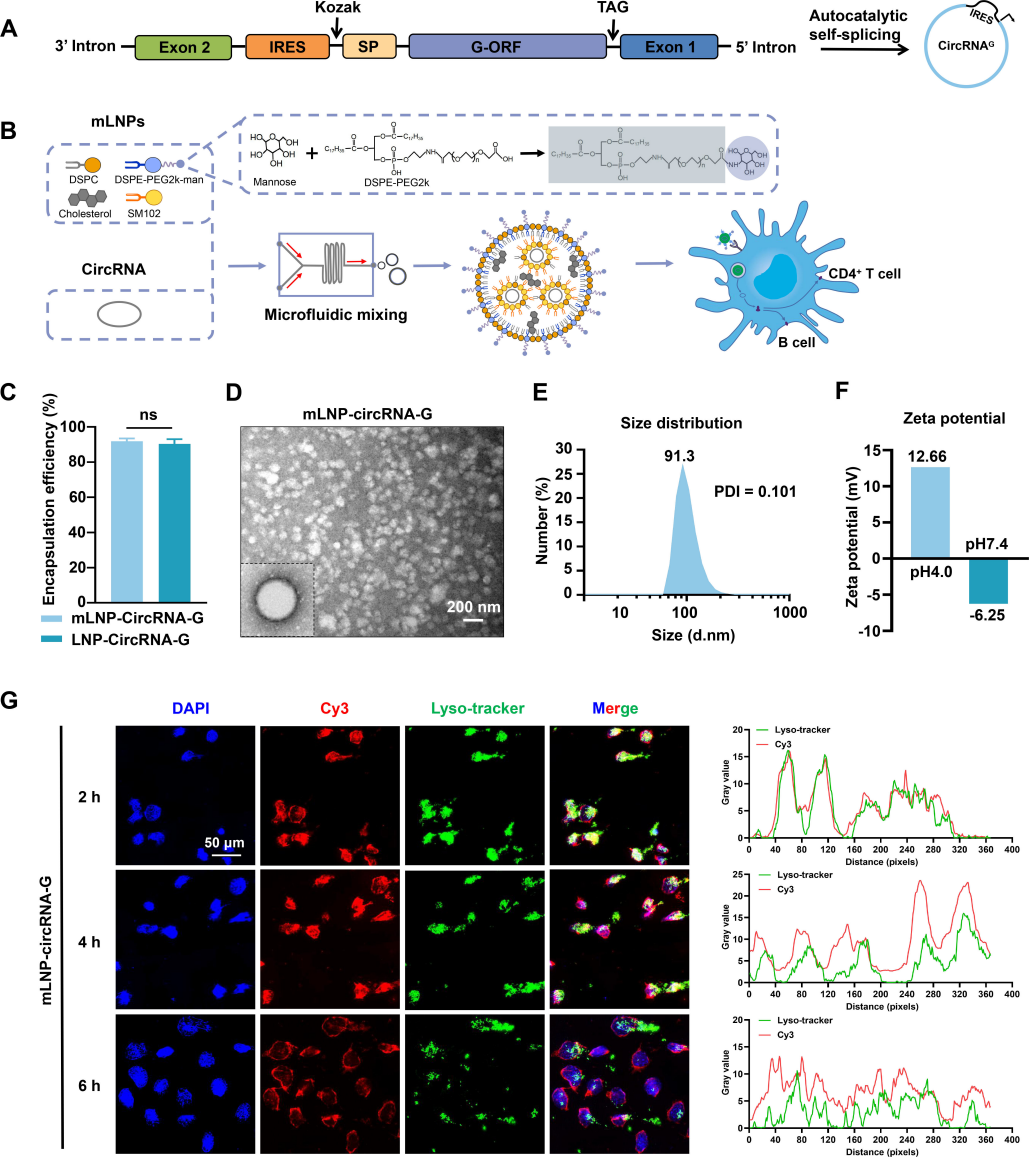
Preparation and characterization of mLNP-circRNA-G. (**A**) Schematic diagram of circRNA-G circularization by group I intron autocatalysis. SP, signal peptide sequence of human tissue plasminogen activator (tPA). IRES, internal ribosome entry site. (**B**) Schematic diagram of the construction of the mLNP-circRNA-G vaccine. (**C**) Encapsulation efficiency of lipid nanoparticles. (**D**) Representative transmission electron microscopy image of mLNPs in solution following circRNA-G encapsulation. Scale bar, 200 nm. (**E**) mLNP particle size by dynamic light scattering. (**F**) Zeta potential of mLNPs at pH 4.0 and 7.4. (**G**) DC2.4 cells were transfected with Cyanine3 (Cy3) fluorescent dye-labeled (red) mLNP-circRNA-G. Intracellular localization of mLNP-circRNA-G (red) and endosomes labeled by LysoTracker Green DND-26 (green) was observed under a confocal microscope at 2, 4, and 6 h. Fluorescence visualization (left) and intensity profiles (right). Scale bar, 50 µm.

Mannose modification is a widespread strategy to enhance nanovaccines uptake by DCs through the mannose receptor (21). We employed the group I intron autocatalysis strategy (7) to produce circular RNAs encoding glycoproteins of the RABV vaccine strain SAD-L16, termed circRNA-G (Fig. 1A). By modifying distearoylphosphatidylethanolamine-polyethylene glycol 2000 (DSPE-PEG2k) with mannose, LNPs were obtained that could target the mannose receptor on the surface of DCs to increase its delivery capacity *in vivo* and *in vitro* (Fig. 1B) (22). mLNP-circRNA-G was produced by squeezing a mixture of alcoholic lipid solution and aqueous circRNA-G solution into the microfluidic chip. The encapsulation efficiency of mannose-modified mLNP-circRNA-G was approximately 92%, which was comparable to that of LNP-circRNA-G, indicating that the encapsulation ability of LNPs was not decreased by mannose modification (Fig. 1C). Transmission electron microscopy (TEM) analysis showed that mLNP-circRNA-G exhibited an electron-dense core (Fig. 1D). The dynamic light scattering of mLNPs in PBS indicated that the average particle size was 91.3 nm (Fig. 1E), whereas particles between 20 and 100 nm efficiently drain through lymphatic vessels into the LNs (23, 24), where they are taken up by LN-resident APCs (25). The narrow polydispersity index (PDI) was 0.101 indicating a homogeneous distribution of mLNPs (Fig. 1E). Zeta potential measurements showed that ionization of the surface charge was observed as the zeta potential increased from −6.25 mV at pH 7.4 to +12.66 mV at pH 4.0 (Fig. 1F). This facilitates escape of the mLNPs from the endosome/lysosome and release of the circRNA into the cytoplasm.

The endosomal escape abilities of mLNP-circRNA-G were further examined *in vitro*. When circRNA vaccines enter cells through the clathrin-mediated or macropinocytosis cellular uptake pathway, circRNA vaccines are transported to endosomes (26). If circRNA vaccines cannot escape from endosomes in time, then they will break down in lysosomes, resulting in low transfection efficiency (27). Cyanine3 (Cy3) fluorescent dye-labeled (red) mLNP-circRNA-G was cocultured with adherent DC2.4 cells. The localization of mLNP-circRNA-G in cells and endosomes labeled with LysoTracker Green DND-26 (green) was observed using fluorescence microscopy at 2, 4, and 6 h post-transfection. Fluorescence imaging illustrated that mLNP-circRNA-G was successfully delivered into the cells. A high yellow fluorescence signal was observed at 2 h, which was the result of the overlap between green fluorescence from endosomes and red fluorescence from mLNP-circRNA-G, suggesting that mLNP-circRNA-G had been absorbed by endosomes. Starting at 4 h, yellow fluorescence began to decrease; it decreased further at 6 h and individual green and red fluorescent signals increased, indicating that mLNP-circRNA-G had escaped from endosomes (Fig. 1G). These results indicate that mLNP-circRNA-G had excellent endosome escape ability.

Furthermore, upon mannose modification of LNPs, we observed that this modification exerted additional effects during the lyophilization of the vaccine. LNPs exhibited significant alterations even with the addition of sucrose during lyophilization. The changes in the physical properties of the nanoparticles (Fig. S1A through D) directly resulted in a decrease in immunogenicity (Fig. S1E and F). Following mannose modification, LNPs demonstrated comparable physical properties to freshly prepared LNPs even after lyophilization (Fig. S1G through J), thereby ensuring the preservation of their immunogenic stability (Fig. S1K and L). These results prompted us to adopt mannose as an LNP-targeting modification strategy and pursue subsequent research.

### Sustained antigen production of circRNA-G induces a durable humoral response

**Fig 2:**
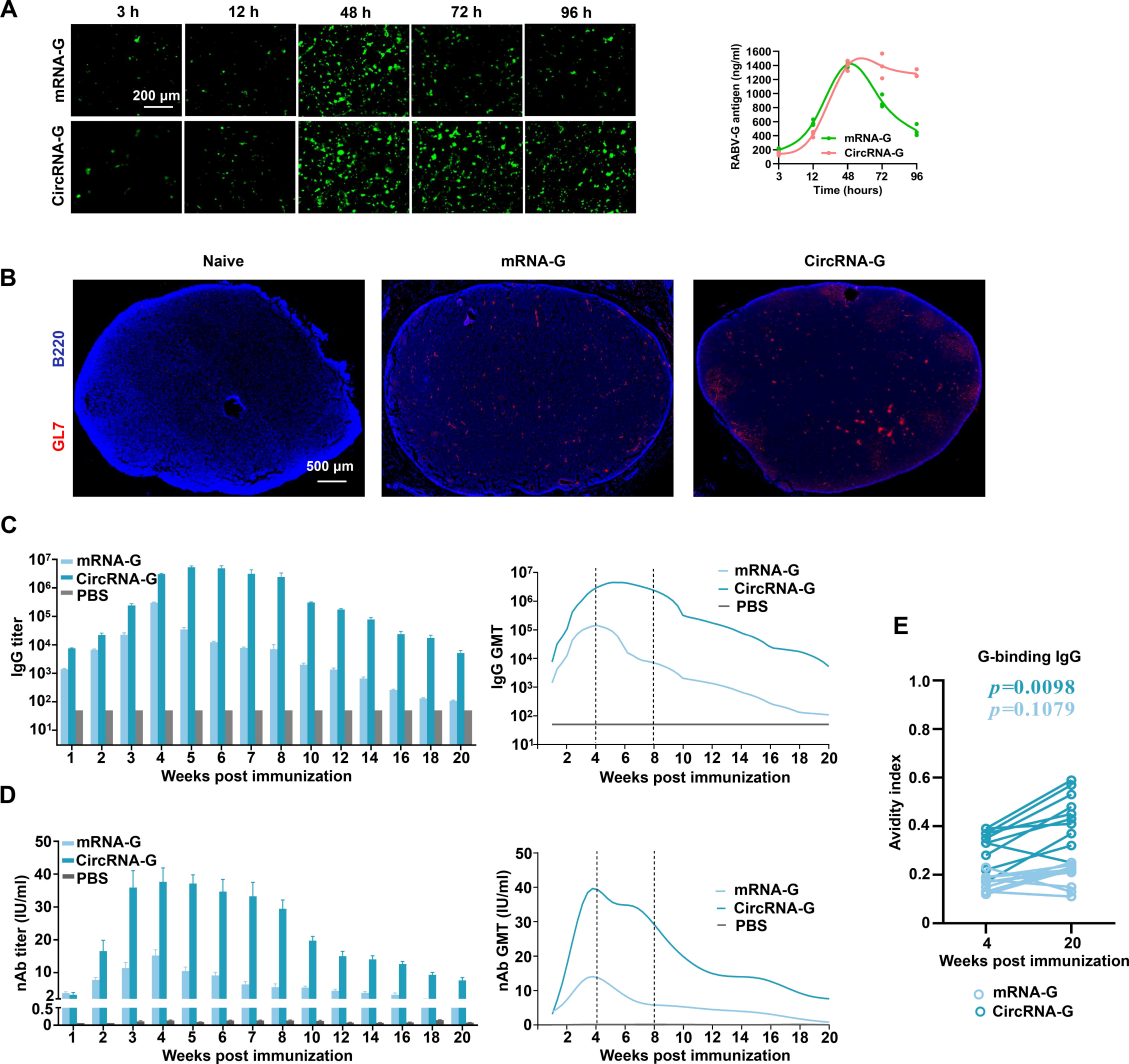
CircRNA-G promotes humoral immune response by prolonging antigen expression. (**A**) CircRNA-G or mRNA-G was transfected into HEK293T cells using jetPRIME. Cells were harvested at selected time points after incubation. GFP expression was visualized using inverted fluorescence microscopy. (**B**) The GC response in the dLNs (day 10) was analyzed by immunofluorescence. LN sections were stained for B cells (B220; blue) and GL7 (red). Scale bar, 500 µm. (**C**) Measurement of the RABV IgG antibody GMTs by ELISA. Error bars indicate means with SEM (*n* = 10). (**D**) nAb titers were measured using FAVN assays. Error bars indicate means with SEM. (**E**) Avidity indices of plasma anti-G IgG.

Distinct from 1mΨ-modified mRNA-G, the high stability of circRNA-G effectively prolonged the time of antigen translation (Fig. S2), which increased the antigen expression of circRNA-G (Fig. 2A). Recent studies have indicated that extended antigen availability improves immune complex formation and, thus, improves the magnitude and quality of GCs (11). To confirm the superior ability of circRNA-G vaccines to foster the formation of GCs after a single immunization, we performed immunofluorescence staining on inguinal LNs from mice immunized 10 days earlier with circRNA-G or mRNA-G. The results showed that circRNA-G induced more GCs on day 10 post-immunization (dpi) (Fig. 2B).

According to the difference in the GC reaction, we next tested whether the circRNA-G vaccine can enhance the humoral response. We administered a single inoculation to mice with 2 µg of circRNA-G or mRNA-G. CircRNA-G elicited a high level of G-specific IgG endpoint geometric mean titers (GMTs), reaching ~5.3 × 10^6^ at 5 weeks post-immunization (wpi) (Fig. 2C), a 396-fold increase in IgG peak times (at 7 wpi) (Fig. S7A) compared to mRNA-G. Similarly, circRNA-G-induced nAb levels reached 37.7 IU/mL at 4 wpi (Fig. 2D), a 9.2-fold increase in nAb peak times (at 20 wpi) compared to mRNA-G (Fig. S7B). Notably, not only did mRNA-G produce lower IgG and nAb than circRNA-G, but also the antibody levels began to decrease significantly after 4 wpi, as expected. Moreover, the nAb titer of mRNA-G was maintained at a low level from 14 wpi, especially at 18 wpi when 20% of the mice were negative for nAb. In contrast, circRNA-G antibody levels peaked at 4 wpi, remained stable from 4 to 8 wpi, and began to decline only after 9 wpi but remained high at 20 wpi (Fig. 2D). Notably, the antibody affinity of circRNA-G was significantly increased compared to that of mRNA-G (Fig. 2E). Together, these data show that circRNA-G immunization generated greater and more persistent humoral responses than mRNA-G immunization.

### LN-targeting delivery optimizes the cellular internalization and biodistribution of mLNP-circRNA-G nanoparticles

**Fig 3:**
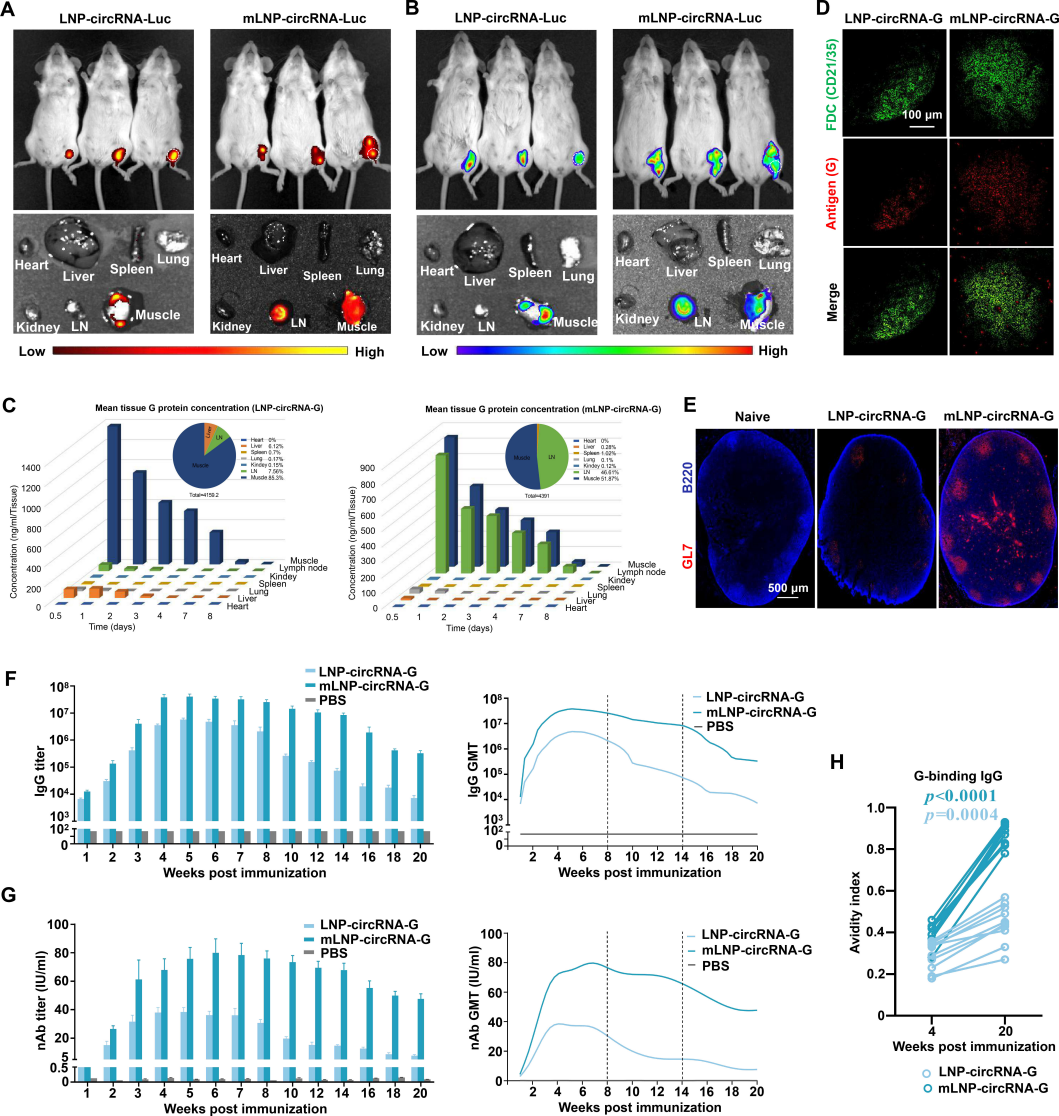
LN-targeting delivery of LNP-circRNA-G enhances humoral immunity. (**A**) *In vivo* imaging system (IVIS) fluorescence images of DiR-labeled mLNP-circRNA-Luc or LNP-circRNA-Luc (containing 5 µg circRNA) 24 h after intramuscular injection (*n* = 3). (**B**) Bioluminescence imaging of luciferase (Luc) expression after mLNP-circRNA-Luc or LNP-circRNA-Luc intramuscular injection in ICR mice. (**C**) Duration and distribution of G protein production from mLNP-circRNA-G or LNP-circRNA-G *in vivo*. Groups of ICR mice (*n* = 3) were vaccinated with one injection of 20 µg of mLNP-circRNA-G or LNP-circRNA-G. Three mice per group were euthanized at selected time points after inoculation. Draining lymph node, muscle, heart, liver, kidney, spleen, and lung samples of each mouse were harvested for quantitation of G protein expression levels by ELISA. Area under the curve (AUC) representing the cumulative distribution of G protein in each specimen (top right corner panel). (**D**) Groups of ICR mice (*n* = 3) were vaccinated with mLNP-circRNA-G and LNP-circRNA-G, as described in A, and lymph nodes were collected for imaging 7 days after vaccination. The FDC network was labeled *in situ* with G protein monoclonal antibody and anti-CD21/35 antibody. Collected tissues were clarified and imaged intact by confocal microscopy. Scale bar, 100 µm. (**E**) The GC response in the dLNs (day 10) was analyzed by immunofluorescence. LN sections were stained for B cells (B220; blue) and GL7 (red). Scale bar, 500 µm. (**F**) Mice were inoculated with a single dose of 2 µg of mLNP-circRNA-G or LNP-circRNA-G by intramuscular injection. Measurement of the RABV IgG antibody GMTs by ELISA. Error bars indicate means with SEM (*n* = 10). (**G**) nAb titers were measured using fluorescent-antibody virus neutralization (FAVN) assays. One-way ANOVA was used to evaluate intergroup differences. Error bars indicate means with SEM (*n* = 10). (**H**) Avidity indices of plasma anti-G IgG (*n* = 10).

The biodistribution of circRNA or mRNA vaccines in tissues after administration may influence safety and efficacy (28). Recent studies have found that targeted delivery to the LN for mRNA vaccines is predicted to increase the immune response (28). To determine the effect of mannose modification on the cellular internalization of LNPs, mLNP-circRNA-G and LNP-circRNA-G were transfected into DC2.4 cells. As shown, a higher proportion of mLNP-circRNA-G was taken up by cells compared to LNP-circRNA-G (Fig. S3A). Higher cellular uptake of mLNP-circRNA-G also led to higher antigen expression (Fig. S3B). To further study the effect of mannose modification on the *in vivo* delivery of LNPs, DIR-labeled mLNP-circRNA-Luc was injected into ICR mice via the hind leg muscles, followed by whole-body fluorescence imaging. It was clearly observed that the mLNPs gradually migrated to the LN, while the LNPs were mainly concentrated at the injection site (Fig. 3A). Moreover, mLNPs persisted in the LNs for at least 5 days (Fig. S4B and C). Unlike LNPs, which are mainly distributed at the injection site, mLNPs are targeted to LNs, and antigen translation is widely distributed at both the injection site and the LNs (Fig. 3B; Fig. S4A).

To further determine the biological distribution of mLNP-circRNA-G *in vivo*, tissues and organs were taken for ELISA analysis. The results showed that the distribution of mLNP-circRNA-G was concentrated at the injection site and in the LNs, and the distribution in the LNs was significantly increased in comparison with that of LNP-circRNA-G (Fig. 3C). Moreover, mLNP-circRNA-G and LNP-circRNA-G demonstrated comparable antigen levels. However, in the LNP-circRNA-G group, only 7.56% of the antigen was localized in the LNs, whereas the proportion of antigen distribution significantly increased to 46.61% in the mLNP-circRNA-G group (Fig. 3C). This indicated that the targeting modification significantly changed the biological distribution of antigen expression. These results indicate that mLNP-circRNA-G preferentially targets LNs and significantly increases biodistribution in the LN. Notably, after modification of the LNPs to target LNs, the proportion of antigen expression in the liver decreased significantly from 6.12% to a low level of 0.28% (Fig. 3C). Therefore, the targeted expression of circRNAs *in vivo* can minimize vaccine accumulation in the liver.

### mLNP-circRNA-G promotes the maturation of DCs *in vitro* and *in vivo*

Mannose modification enhances DC targeting *in vivo* and *in vitro* and can promote DC maturation (14, 29). Next, we evaluated the ability of mLNP-circRNA-G to promote DC maturation. Bone marrow-derived DCs (BMDCs) were prepared and treated with different concentrations of mLNP-circRNA-G or LNP-circRNA-G for 24 h. Then, mature BMDCs (CD11c^+^CD86^+^ cells, CD11c^+^CD80^+^ cells, and CD11c^+^MHCII^+^ cells) were analyzed by flow cytometry (Fig. S5A and S6A, C, E). Both mLNP-circRNA-G and LNP-circRNA-G treatment promoted DC maturation, but significantly higher proportions of CD11c^+^CD86^+^ cells, CD11c^+^CD86^+^ cells, and CD11c^+^CD86^+^ cells were observed in BMDCs treated with mLNP-circRNA-G than in those treated with LNP-circRNA-G (Fig. S6B, D, and F).

Previous studies have established that mature DCs can enhance rabies vaccine-induced humoral immune responses (30–32). Next, we investigated the effect of mLNP-circRNA-G on DC maturation in mice *in vivo*. Three groups of C57BL/6 mice were immunized with 2 µg of mLNP-circRNA-G or LNP-circRNA-G by the intramuscular (i.m.) route. Inguinal LNs were collected at 48 hpi, single-cell suspensions were prepared, and CD11c^+^CD80^+^ and CD11c^+^CD86^+^ cells were analyzed by flow cytometry (Fig. S5B and S6G). Significantly more mature DCs were observed in the LNs of mice immunized with mLNP-circRNA than in those of mice immunized with LNP-circRNA (Fig. S6H). These results suggest that mLNP-circRNA can effectively promote the maturation of DCs *in vitro* and *in vivo*.

### LN-targeting delivery results in prolonged antigen retention in LNs and enhanced GC formation and antibody responses

The successful targeting of LNs by mLNP-circRNA-G nanoparticles, together with their enhanced and sustained activation of DCs *in vitro* and *in vivo*, encouraged us to assess the subsequent effects on the humoral immune response, which largely depend on the GC reaction (33). To better evaluate the effect of targeted LNs on antibody production, we immunized mice with 2 µg of mLNP-circRNA-G and LNP-circRNA-G vaccines by intramuscular injection. To determine the anatomical localization of retained antigen, we stained the LNs with monoclonal antibodies against RABV-G protein and anti-CD21/35 antibody (surface marker for follicular dendritic cells). Twenty-four hours after LNP-circRNA-G vaccine immunization, little or no antigen could be detected on follicular dendritic cells (FDCs) or any other location in LNs, whereas 24 h after mLNP-circRNA-G vaccine immunization, substantial amounts of RABV-G were detected lining the FDC network (Fig. 3D). Thus, target LN delivery leads to enhanced antigen retention within LNs, with preferential retention on FDCs.

We next analyzed GC formation in the inguinal dLNs of mice receiving LNP-circRNA-G vs mLNP-circRNA-G vaccine immunization. We first performed immunofluorescence staining of dLNs from mice immunized with LNP-mRNA-G or mLNP-circRNA-G. The results showed that mLNP-circRNA-G induced more GCs at 10 dpi (Fig. 3E). Next, we wanted to know whether the retention of antigen in LNs and the enhancement of GC formation have an obvious impact on antibody production. We immunized mice with LNP-circRNA-G or mLNP-circRNA-G and examined antibodies against RABV-G post-immunization. The IgG and nAb titers of the mLNP-circRNA-G group remained stable from 4 to 14 wpi of the priming phase in the absence of booster immunization (Fig. 3F and G). Notably, all mice receiving mLNP-circRNA-G immunization generated robust nAb responses after the prime immunization. mLNP-circRNA-G-inoculated mice had IgG GMTs reaching ~4.1 × 10^7^ at 5 wpi, a 116-fold (Fig. S7C) increase in IgG peak times (at 14 wpi) compared to LNP-circRNA-G. mLNP-circRNA-G-inoculated mice had nAb titers up to 80 IU/mL at 6 wpi, a 6.3-fold (Fig. S7D) increase in nAb peak times (at 20 wpi) compared to those obtained with LNP-circRNA-G. Moreover, mLNP-circRNA-G antibody affinity was significantly increased compared to LNP-circRNA-G (Fig. 3H). Additionally, mLNP-circRNA-G elicited IgG2a, IgG2b, and IgG1 subclass G-specific antibodies, indicating a balanced Th1/Th2 response (Fig. S8). Together, these results highlight the importance of LN-targeting delivery in the generation of a potent and durable adaptive immune response after immunization.

### mLNP-circRNA-G promotes the generation of Tfh cells, activated B cells, GC B cells, LLPCs, and RABV-specific ASCs

**Fig 4:**
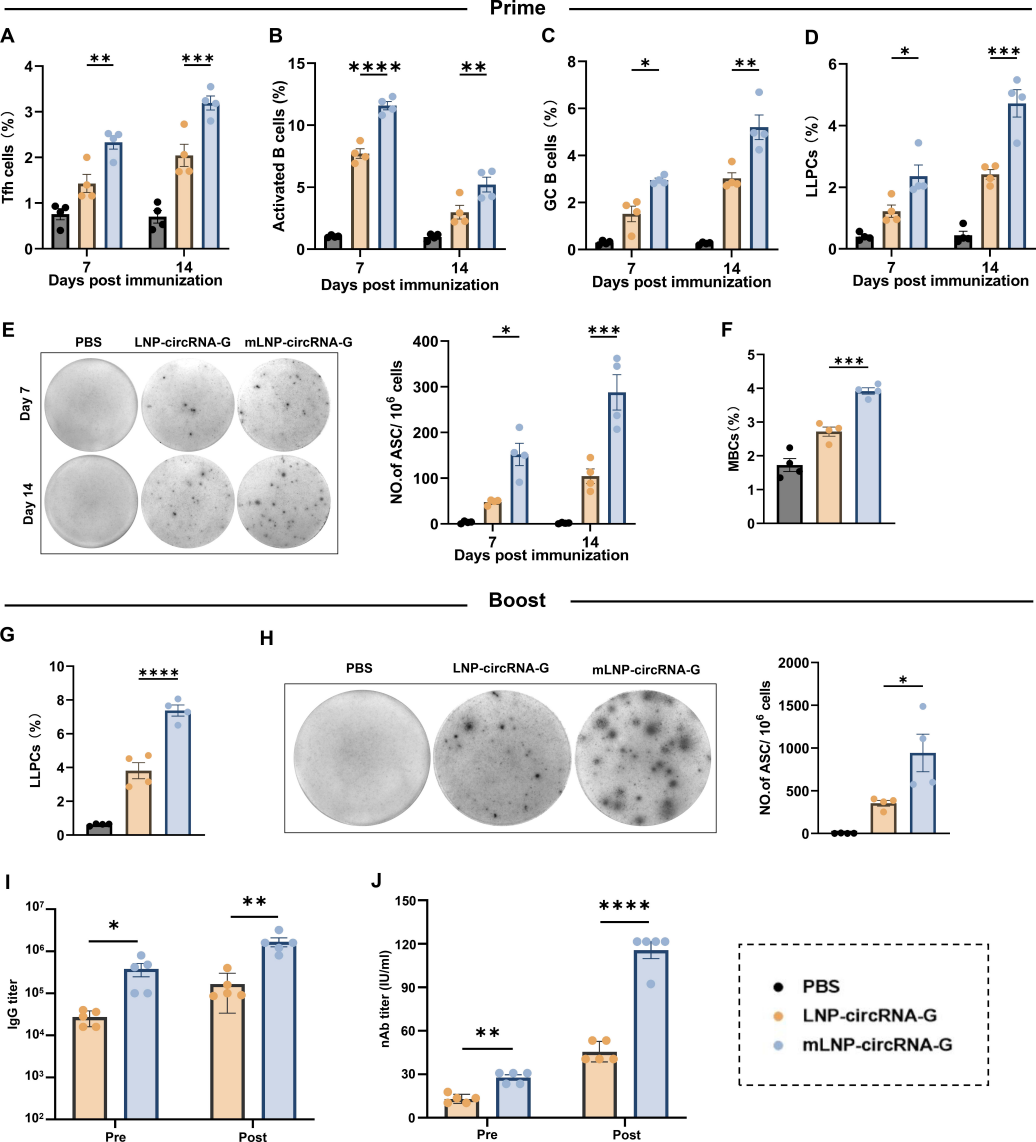
mLNP-circRNA-G facilitates the generation of Tfh cells, activated B cells, GC B cells, long-lived plasma cells (LLPCs), RABV-specific ASCs, memory B cells (MBCs), and secondary antibody responses. (**A–D**) C57BL/6 mice were immunized with 2 µg of mLNP-circRNA-G, LNP-circRNA-G, or DMEM. Draining LNs or BMs were collected at 7 and 14 dpi, and cell suspensions were analyzed by flow cytometry. Statistical results of Tfh cells (**A**), activated B cells (**B**), GC B cells (**C**), and LLPCs (**D**). Error bars represent SEM (*n* = 4). (**E**) Representative sections of the ELISpot assay and the statistical results of ASCs. Single inguinal LN cells were prepared for calculating RABV-specific ASCs by ELISpot assay at 7 and 14 dpi. (**F**) Statistical results of MBCs. C57BL/6 mice (*n* = 4) were immunized with 2 µg of mLNP-circRNA-G, LNP-circRNA-G, or DMEM. At 100 dpi, single LN cells were stained with antibodies to analyze the MBCs by flow cytometry. (**G**) Statistical results of LLPCs. C57BL/6 mice (*n* = 4) were boosted with 2 µg of mLNP-circRNA-G or LNP-circRNA-G at 100 days after primary immunization. At 14 days after the booster, LLPCs in BMs were evaluated by flow cytometry. (**H**) Representative sections of the ELISpot assay and the statistical results of ASCs. RABV-specific ASCs in inguinal LNs were evaluated by ELISpot assay 14 days after the booster. (**I and J**) RABV-specific IgG (**I**) and nAb (**J**) titers were evaluated 14 days after secondary immunization. Error bars represent SEM (*n* = 5).

Humoral immune responses mediated by CD4^+^ T cells are essential for RABV elimination. T follicular helper (Tfh) cells are a subset of CD4^+^ T cells specialized in regulating affinity maturation of B cells in GCs (34). Therefore, we investigated the role of mLNP-circRNA-G in the induction of Tfh cell proliferation. C57BL/6 mice were immunized with 2 µg of mLNP-circRNA-G, LNP-circRNA-G, or DMEM, and Tfh cells (CD4^+^PD1^+^CXCR5^+^) were quantified at 7 and 14 dpi (Fig. S9A and S10A). At 7 and 14 dpi, more Tfh cells were observed in the mice immunized with mLNP-circRNA-G than in the mice immunized with LNP-circRNA-G (Fig. 4A). In addition, we evaluated activated B cells (B220^+^MHCII^+^CD86^+^) and GC B cells (B220^+^GL7^+^CD95^+^) at 7 and 14 dpi (Fig. S9B; Fig. S10B and C). The results indicated that mLNP-circRNA-G induced more activated B-cell and GC B-cell production than LNP-circRNA-G in mice (Fig. 4B and C).

Long-lived plasma cells (LLPCs) persistently secrete Abs without the need for antigen re-exposure and are responsible for maintaining levels of circulating Abs that can prevent or hinder reinfection by pathogens (35, 36). We were able to measure strong Tfh cell, GC B-cell responses, and durable Ab responses after a single immunization with mLNP-circRNA-G vaccines. Next, we evaluated the effect of mLNP-circRNA-G on LLPC formation. Mice were vaccinated with 2 µg of mLNP-circRNA-G, LNP-circRNA-G, or DMEM, and the percentage of LLPCs in the bone marrow (BM) was assessed by flow cytometry at 7 and 14 dpi (Fig. S9C and S10D). The results showed that mice immunized with mLNP-circRNA-G produced significantly more LLPCs in BM than mice immunized with LNP-circRNA-G (Fig. 4D). In addition, the generation of RABV-specific antibody-secreting cells (ASCs) in LNs was assessed by ELISpot assay at 7 and 14 dpi. As expected, mice immunized with mLNP-circRNA-G possessed significantly more RABV-specific ASCs than mice immunized with LNP-circRNA-G (Fig. 4E). These data suggest that mLNP-circRNA-G is more effective than LNP-circRNA-G in promoting the long-term humoral immune response.

### mLNP-circRNA-G induces a strong secondary antibody response

Long-term humoral immune protection against pathogen infection relies on LLPCs and memory B cells (MBCs) (37). MBCs are the second line of defense upon infection and can be rapidly reactivated and generate a strong antibody response. To assess whether mLNP-circRNA-G can induce immune memory, we analyzed the proportion of MBCs (B220^+^CD38^+^CD138^−^) by flow cytometry after initial immunization (Fig. S11). At 100 dpi, mLNP-circRNA-G-immunized mice exhibited significantly more MBCs than LNP-circRNA-G-immunized mice (Fig. 4F; Fig. S12A). To further assess the effect of mLNP-circRNA-G on secondary antibody responses, mice were boosted with mLNP-circRNA-G or LNP-circRNA-G at 100 dpi. Two weeks after secondary immunization, LLPC (B220^low^CD138^+^) in BMs and RABV-specific ASCs in LNs were assessed by flow cytometry and ELISpot assays, respectively. mLNP-circRNA-G induced significantly more LLPCs (Fig. 4G; Fig. S12B) and RABV-specific ASCs than LNP-circRNA-G (Fig. 4H). Correspondingly, mLNP-circRNA-G induced higher RABV-specific IgG (Fig. 4I) and nAb (Fig. 4J) levels than LNP-circRNA-G in mice on day 14 after booster immunization. Together, these results suggest that mLNP-circRNA-G induces more MBCs and better secondary immune responses than LNP-circRNA-G.

### A single-dose mLNP-circRNA-G vaccine protects mice against virulent RABV challenge

**Fig 5:**
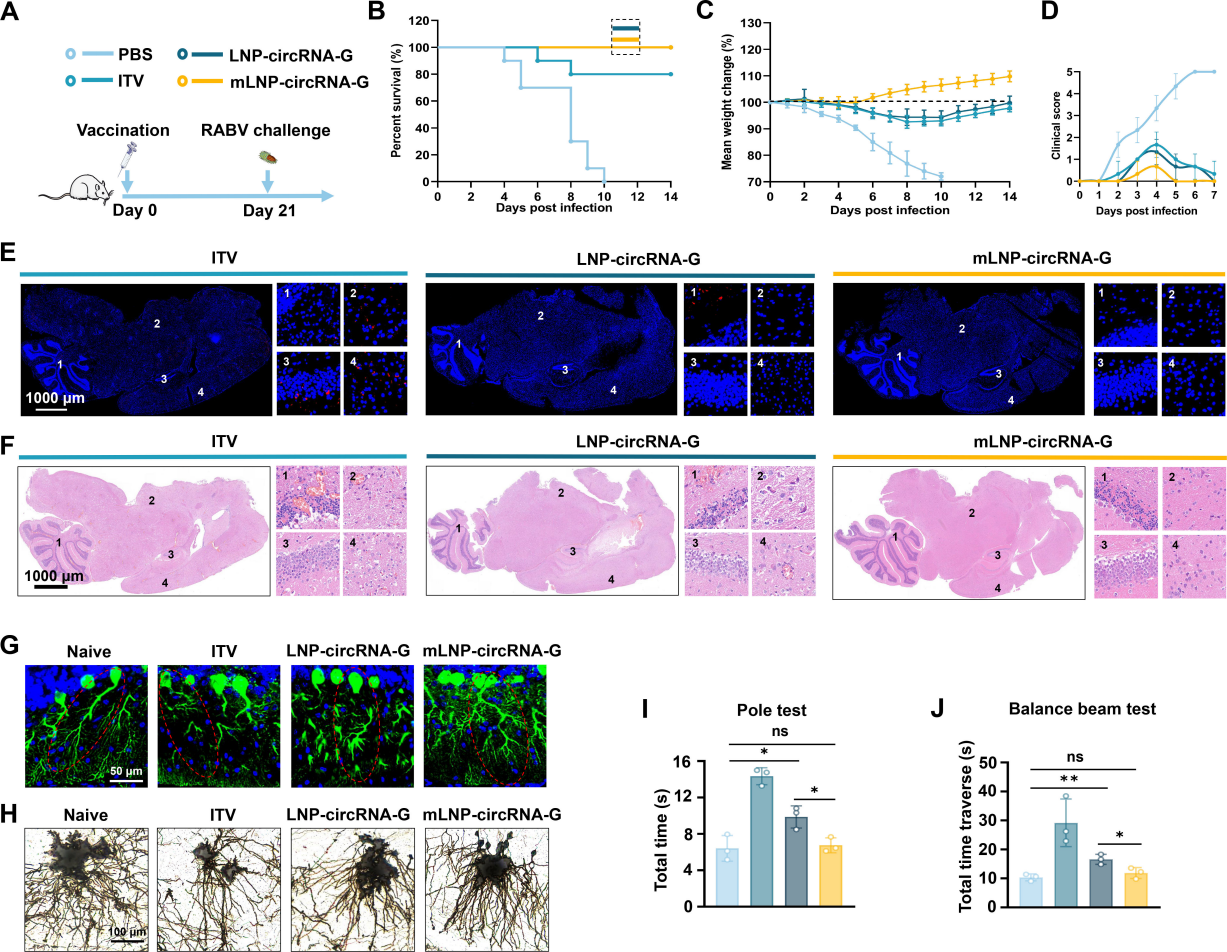
mLNP-circRNA-G vaccine protects mice against RABV. (**A**) Scheme of mLNP-circRNA-G immunization and RABV challenge. Each mouse received intramuscular injections of 2 µg of mLNP-circRNA-G or LNP-circRNA-G followed by intracranial injection of 50 LD_50_ of the RABV strain, CVS-24. Mice receiving a 0.1 dose of a commercial inactivated vaccine, were used as positive controls. (**B**) Survival rates. A log-rank test was used to evaluate intergroup differences in survival rates. (**C**) The body weight changes of mice. (**D**) The clinical scores of mice. (**E**) Viral level analysis of brain sections. Representative images of RABV detected using P protein monoclonal antibody. (**F**) Histological analysis of brain sections. Brains were sectioned and stained with H&E. (**G**) Representative images of immunostaining for Purkinje cells (in green). Immunofluorescence staining for calbindin antibody in brain sections prepared 7 days after viral infection. Scale bars, 50 µm. (**H**) Representative images of Golgi staining for neurocytes. (**I and J**) The impact of viral infection on spatial memory and motor ability was assessed using animal behavior tests. In mice that survived the virus challenge, sequelae affecting the nervous system were assessed. At 7 days after viral infection, mice were evaluated using the climbing test (**I**) and balance beam test (**J**).

To further explore the protective effect of the mLNP-circRNA-G vaccine against RABV infection *in vivo*, ICR mice were immunized with a single dose of 2 µg of mLNP-circRNA-G, 2 µg of LNP-circRNA-G, or a 0.1 dose of inactivated rabies vaccine (ITV) via the muscular route and challenged with a lethal dose of 50 LD_50_ of the virulent RABV strain CVS-24 after week 3 post-immunization (Fig. 5A). We observed 100% survival in mice administered the mLNP-circRNA-G or LNP-circRNA-G vaccine and 80% survival in mice administered the ITV. In addition, all PBS-inoculated mice succumbed to rabies within 10 days (Fig. 5B). Stable increases in body weight after transient decreases were observed in the LNP-circRNA-G vaccine and ITV groups. Moreover, the body weight change and clinical scores showed that the mLNP-circRNA-G vaccine group was least affected by viral infection (Fig. 5C and D). Immunofluorescence staining of brain tissue showed that all mice inoculated with ITV were infected. The virus was essentially undetectable in the brains of the mLNP-circRNA-G vaccine group, while low levels were detected of the brain of the LNP-circRNA-G group (Fig. 5E). The results showed that inoculation with the mLNP-circRNA-G vaccine effectively inhibited virus invasion into mouse brains.

Further histopathological examination demonstrated that all mLNP-circRNA-G- and LNP-circRNA-G-immunized mice were well protected, as only very mild pathological damage was observed in the LNP-circRNA-G group, and almost no pathological damage was observed in the mLNP-circRNA-G group. In contrast, more lesions, such as local neuronal necrosis and degeneration, neurophagocytic phenomena and a large number of inflammatory cell infiltrates, were observed in the brains of the ITV group (Fig. 5F). Through further staining of neurons, we found that the dendritic complexity of mouse neurons in the ITV group was significantly reduced, and the length was reduced. There was also a slight reduction in dendritic complexity in the LNP-circRNA-G vaccine group. Consistently, there were no differences between mLNP-circRNA-G-treated mice and normal mice (Fig. 5G and H). The climbing pole test and balance beam test are effective methods for assessing learning and motor balance in mice. Mice in all groups were similarly affected by viral infection, except that there were no differences between the mLNP-circRNA-G group and the healthy group mice (Fig. 5I and J). These data demonstrated that a single vaccination of mLNP-circRNA-G afforded remarkable prevention of RABV replication in the brain and efficiently protected mice from brain lesions. Notably, mice vaccinated with LNP-circRNA-G were still affected by viral infection in a short time after vaccination.

### Duration and long-term protection induced by single-dose mLNP-circRNA-G

**Fig 6:**
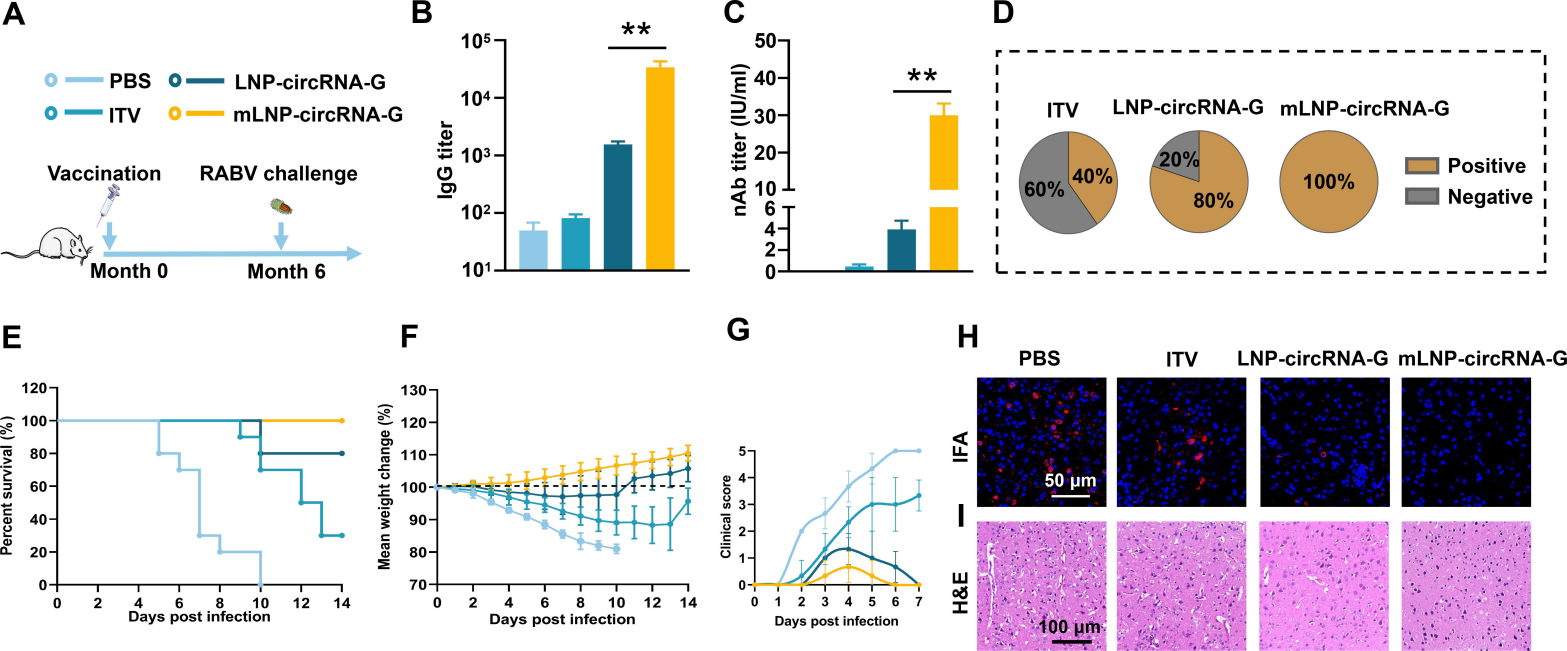
Long-term protective efficacy of mLNP-circRNA-G against RABV in mice. (**A**) Scheme of mLNP-circRNA-G immunization and RABV challenge. Each mouse received intramuscular injections of 2 µg of mLNP-circRNA-G or LNP-circRNA-G followed by intracranial injection of 50 LD_50_ of the RABV strain CVS-24 6 months after inoculation. Mice receiving a 0.1 dose of commercial inactivated vaccine were used as positive controls (*n* = 10). (**B**) Measurement of the IgG antibody titer elicited by the vaccine. (**C**) Measurement of the nAb titer elicited by the vaccine. (**D**) The proportion of mice with seroconversion induced by vaccination. nAb values greater than 0.5 IU/mL were considered positive. (**E**) Survival rates. A log-rank test was used to evaluate intergroup differences in survival rates. (**F**) Mouse body weight changes. (**G**) The clinical scores of mice. (**H and I**) Histological and viral level analysis of brain sections. Representative images of RABV detected using P protein monoclonal antibodies (**H**). Brains were sectioned and stained with H&E (**I**).

To evaluate the durability and long-term protection via the antibody response elicited by a single mLNP-circRNA-G immunization, we performed a viral challenge study at month 6 after immunization. Mice were inoculated as in the viral attack assay described above, and serum was collected at month 6 after inoculation for analysis of the antibody response and subjected to viral attack (Fig. 6A). The results showed that the IgG and nAb titers remained at ~4.6 × 10^4^ and ~30 IU/mL, respectively, 6 months after inoculation with the mLNP-circRNA-G vaccine (Fig. 6B and C). The statistical findings on the seroconversion rate of nAb were notable. At 6 months after inoculation, the seroconversion rate remained at 40% and 80% for the inactivated and LNP-circRNA-G vaccines, respectively, meaning that 60% and 20% of their mice became negative for nAb, respectively. Seroconversion was defined as antibody titers ≥0.5 IU/mL, which is the threshold defined by the WHO (38). All mice inoculated with the mLNP-circRNA-G vaccine were positive for nAb (Fig. 6D).

One day after the collection of blood, mice were infected with 50 LD_50_ RABV via the intracranial route. We observed that 100% of mice vaccinated with mLNP-circRNA-G remained alive, while the survival rate in the LNP-circRNA-G group was 80%. Notably, the survival rate of mice in the ITV group was only 30% (Fig. 6E). These results differ significantly from the challenge test conducted on mice 3 weeks after vaccination, with the mice vaccinated with mLNP-circRNA-G exhibiting a 100% survival rate, while both the LNP-circRNA and ITV groups experienced a substantial decrease in survival rates. Antibody and survival data further suggested that although ITV and LNP-circRNA-G showed high protective efficacy shortly after inoculation, the duration of immunoprotection was short. In contrast, a single immunization with mLNP-circRNA-G elicited a long-term antibody response and protection. Body weight changes and clinical scores showed that the mLNP-circRNA-G vaccine group was minimally affected by viral infection (Fig. 6F and G). Viral fluorescence staining of brain tissue showed that mice in the LNP-circRNA-G group were infected. The virus was still not detected in the brains of the mLNP-circRNA-G vaccine group (Fig. 6H and I). Together, these data suggest that a single immunization with mLNP-circRNA-G conferred long-term protection against RABV infection by eliciting a durable humoral response.

### Lyophilized mLNP-circRNA-G exhibits LN-targeting stability and potent immunogenicity

It has been demonstrated that mLNP-circRNA-G can induce potent and persistent immune responses, but one of the prerequisites of the wide application of circRNA vaccines is overcoming the challenge of long-term storage of mLNP-circRNA vaccines (39). We and others have demonstrated that using LNPs with targeting modifications to deliver mRNA to specific organs and cells can effectively improve the immune response (40–42). To determine whether the lyophilization process will affect the targeting modification of mLNPs and, thus, the immunogenicity, we lyophilized mLNP-circRNA-G and then stored the lyophilized mLNP-circRNA-G vaccine at 4°C for 24 weeks. The lyophilized mLNP-circRNA-G exhibited a round cake-like morphology, was arranged in dense particles under a scanning electron microscope (SEM) (Fig. S13A and B), and dissolved easily and rapidly in water. The rehydrated solution is homogeneous and translucent, and there is no obvious difference from the freshly prepared mRNA-LNP solution (Fig. S13C). Moreover, the nanomorphology was well maintained (Fig. S13D). The size (Fig. S13E), PDI (Fig. S13F), zeta potential (Fig. S13G), and encapsulation efficiency (Fig. S13H) of mLNP-circRNA-G showed no significant changes, indicating that the lyophilization process did not change their basic physical properties.

**Fig 7:**
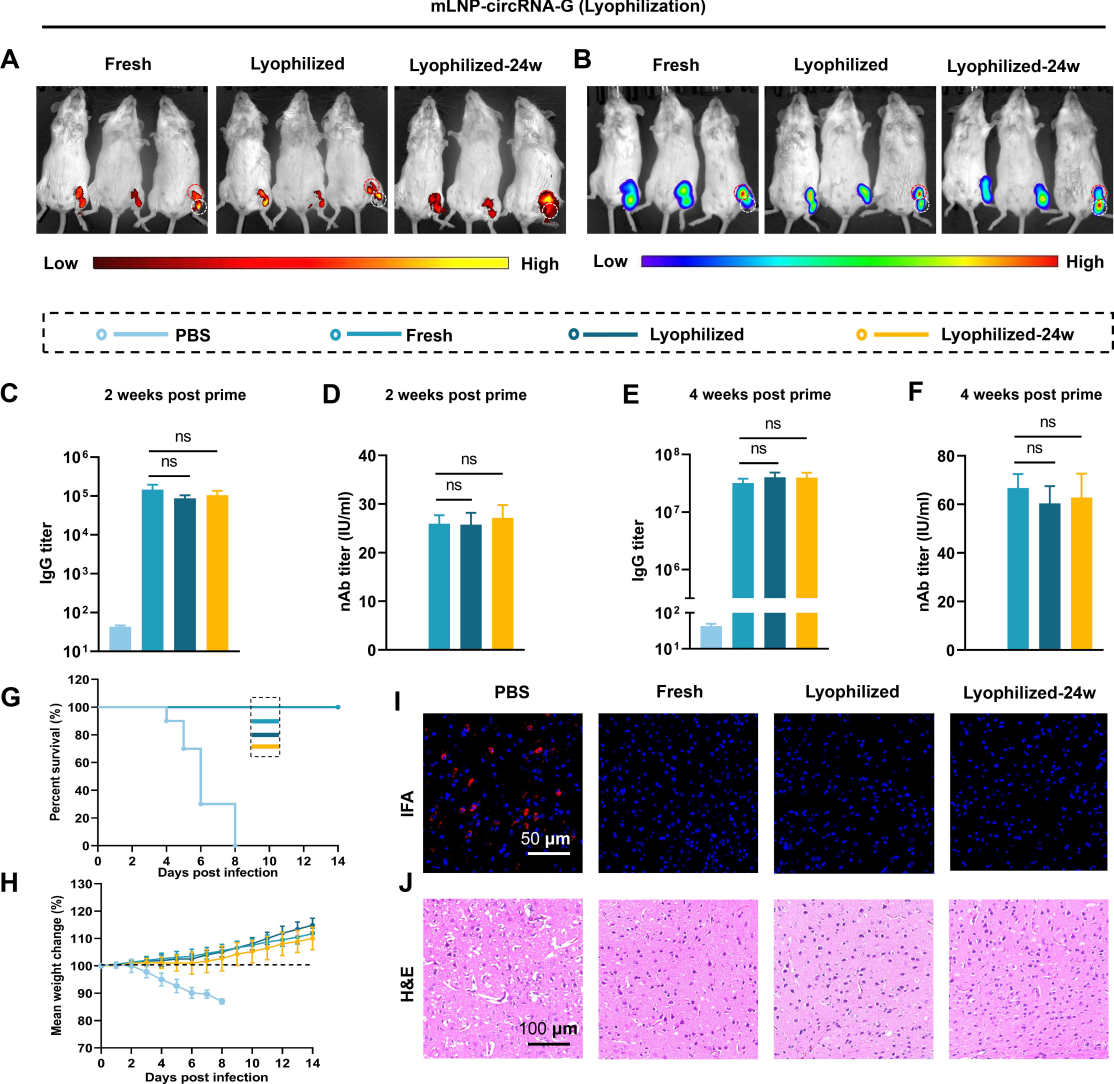
Lyophilized mLNP-circRNA-G remains a target for delivery and potent immunogenicity. (**A**) Lyophilized mLNP-circRNA-Luc was stored at 4 ℃ for 24 weeks. Fluorescence images of DiR-labeled mLNP-circRNA-Luc (containing 5 µg circRNA) 24 h after intramuscular injection (*n* = 3). (**B**) Bioluminescence imaging of mice 24 h after intramuscular injection with freshly prepared or lyophilized mLNP-circRNA-Luc. (**C–F**) Mice were inoculated with a single dose of 2 µg of freshly prepared or lyophilized mLNP-circRNA-G by intramuscular injection, and the IgG titers (**C and E**) and nAb titers (**D and F**) of serum harvested in week 2 and week 4 were measured. (**G and H**) Survival rates (**G**) and body weight changes (**H**) of mice. (**I and J**) Histological and viral level analysis of brain sections. Representative images of RABV detected using RABV P protein monoclonal antibody (**I**). Brains were sectioned and stained with H&E (**J**).

Groups of mice were immunized with a single dose of 2 µg of freshly prepared and lyophilized mLNP-circRNA-G or mLNP-circRNA-Luc. The results showed that the lyophilized mLNPs and the lyophilized mLNPs stored for 24 weeks both targeted LNs with similar effectiveness to the freshly prepared mLNPs, indicating that targeting remained stable after lyophilization (Fig. 7A and B). Moreover, the immunogenicity of the lyophilized vaccine did not decrease significantly within 24 weeks. (Fig. 7C through F). In the challenge test, there was no difference between the lyophilized vaccine and the freshly prepared vaccine (Fig. 7G through J). In summary, the lyophilized mLNPs maintained satisfactory LN targeting and immunogenicity, and the thermostability was significantly improved. These results show that the one-step preparation of mLNPs after mannose modification of PEG lipids is beneficial for maintaining the targeting stability of the mLNP-circRNA vaccine during lyophilization. The stability of targeting also maintains the immunogenicity.

### A SARS-CoV-2 trimeric RBD circRNA vaccine based on mLNPs elicits potent and stable immunogenicity after lyophilization

**Fig 8:**
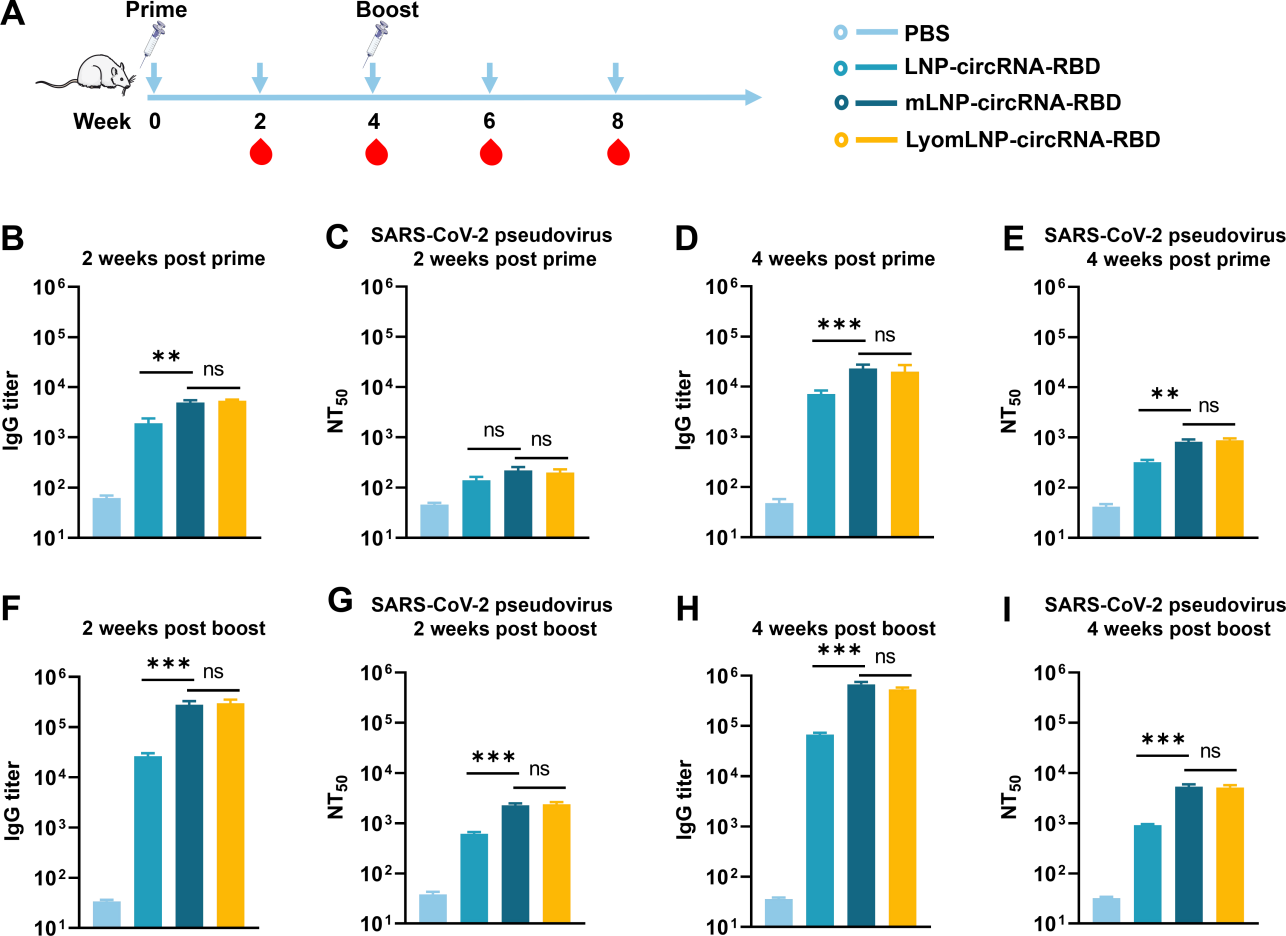
Humoral immune response of the SARS-CoV-2 mLNP-circRNA-RBD vaccine in mice. (**A**) Scheme of mLNP-circRNA-RBD immunization. Each mouse was injected with 5 µg of mLNP-circRNA-RBD, LNP-circRNA-RBD and lyophilized mLNP-circRNA-RBD (LyomLNP-circRNA-RBD) by intramuscular injection, and serum was collected for IgG and NT_50_ analysis at week 2 and week 4 after primary immunization and booster immunization, respectively (*n* = 5). (**B–E**) Serum SARS-CoV-2 RBD protein-binding IgG titer (**B and D**) and serum SARS-CoV-2 neutralizing antibodies against the original Wuhan strain (**C and E**) of mice after primary immunization. (**F–I**) IgG titer (**F and H**) and NT_50_ (**G and I**) of mice after booster immunization.

SARS-CoV-2 infection is a serious threat to human health worldwide, and vaccines against SARS-CoV-2 are critical (43, 44). To demonstrate that the mLNP-circRNA strategy can improve the immunogenicity of other antigens, we used mLNPs to test the SARS-CoV-2 trimeric RBD circRNA vaccine in mice (Fig. 8A). We first evaluated SARS-CoV-2 RBD-specific IgG by ELISA. The results showed that the IgG titer in mLNP-circRNA-RBD was significantly higher than that in LNP-circRNA-RBD in week 2 and week 4 after primary and booster immunization (Fig. 8B, D, F, and H). After further statistical analysis, from week 2 to week 4 after the primary immunization, the IgG titer of mLNP-circRNA-RBD increased by 4.6-fold, and that of LNP-circRNA-RBD increased by 3.7-fold (Fig. 8B and D). In week 2 after booster immunization, mLNP-circRNA-RBD increased 12.2-fold and that of LNP-circRNA-RBD increased 3.7-fold (Fig. 8D and F). Additionally, mLNP-circRNA-RBD elicited IgG2a and IgG1 subclass RBD-specific antibodies, indicating a balanced Th1/Th2 response (Fig. S14).

Furthermore, we used a pseudovirus neutralization assay to assess anti-SARS-CoV-2-nAbs in the serum of vaccinated mice. The pseudovirus neutralization assay showed that the mean NT_50_ (50% neutralization titers) of mLNP-circRNA-RBD and LNP-circRNA-RBD did not differ at week 2 after primary immunization. At week 4, the NT_50_ value of mLNP-circRNA-RBD was significantly higher than that of LNP-circRNA-RBD in week 4, reaching 820, which was 2.6-fold that of LNP-circRNA-RBD (Fig. 8C and E). Notably, in week 2 after booster immunization, the NT_50_ of mLNP-circRNA-RBD increased by 2.8-fold and that of LNP-circRNA-RBD increased by 1.9-fold (Fig. 8E and G). In week 4 after booster immunization, the increase in NT_50_ for mLNP-circRNA-RBD remains greater than that for LNP-circRNA-RBD (Fig. 8I). Comparing the growth of IgG and NT_50_ after booster immunization, mLNP-circRNA-RBD elicited a stronger immune response than LNP-circRNA-RBD. Moreover, the lyophilized mLNP-circRNA-RBD has elicited comparable antibody levels to the freshly prepared mLNP-circRNA-RBD. These results indicated that the delivery of circRNA-RBD by mLNPs significantly improved the antibody response.

### Safety and stability profiles of mLNP-circRNA-G

Although efficacy is critical for the development of vaccines, safety is undoubtedly more important. To further evaluate the safety of mLNP-circRNA-G vaccines *in vitro* and *in vivo*, physiological and biochemical indicators were monitored. Cytotoxicity analysis of mLNP-circRNA-G in HEK-293T cells was performed using MTT assays (Fig. S15A). No severe clinical adverse effects were observed following the high-dose (30 µg) inoculation. The body weights of mice in the vaccination group and the normal control group increased steadily within the normal range (Fig. S15B). After 24 h, serum samples were collected for biochemical analysis. No significant changes in biochemical indicators [urinary anhydride (UREA), total protein (TP), aspartic acid aminotransferase (AST), and alanine aminotransferase (ALT)] were observed in mLNP-circRNA-G- and mLNP-vaccinated mice compared to mice in the PBS group (Fig. S15C). There was no significant difference in other biochemical indicators (Fig. S16). After vaccination, the tissues were isolated and examined via H&E staining. The images showed no noticeable histopathological differences in the main organs between the groups treated with mLNP-circRNA-G or mLNPs and the PBS group (Fig. S15D). Because of the absence of accumulation of LNPs in the liver, no liver damage was observed in mLNP-circRNA-G-treated mice. In summary, our study provides preliminary proof of safety for mLNP-circRNA-G vaccination in mice.

Moreover, the stability of circRNA vaccines plays a crucial role in their storage and transportation. To assess vaccine stability, mLNP-circRNA-G was stored at 4°C for different durations (0, 7, 14, and 28 days). The changes in particle size, zeta potential, encapsulation efficiency, and antibody titer were evaluated. Notably, no significant changes were observed in the particle size, zeta potential, encapsulation efficiency, or IgG titer after storage at 4°C for different time intervals. These results highlight the excellent short-term stability of mLNP-circRNA-G after preparation (Fig. S17).

## DISCUSSION

mRNA vaccines are the key technology to combat existing and emerging infectious diseases. However, increasing the potency, stability, and durability of the vaccine response remains a challenge (45). Moreover, the crucial fact is that for most viral vaccines, the induced immune protection cycle is generally short, which leads to the need for frequent vaccination to provide long-term immune protection. Here, we report a general strategy to enhance the durability and magnitude of antibody responses elicited by vaccination, accomplished by targeting the delivery of a circRNA vaccine to promote draining LN accumulation and produce a stronger and persistent stimulatory effect. Overall, our study revealed the superior ability of the targeting-LN delivery circRNA vaccines to elicit GC responses, which were associated with efficient production of nAbs after a single immunization. Moreover, circRNA vaccines efficiently promoted strong Tfh cells, GC B cells, and durable Ab responses. Our data highlight the importance of LN-targeting delivery for the generation of durable Abs and suggest mLNP-circRNA vaccines as promising candidates to elicit long-term and high-quality adaptive immune responses. We and others have demonstrated that immunogenicity can be improved by targeting specific organs or cells (46). Notably, we verified that the mLNP-circRNA vaccine still has long-term targeting stability and immunogenicity after lyophilization. The mLNP-circRNA vaccine presented in this study not only holds great potential for developing more potent vaccines against RABV and SARS-CoV-2 but also provides a useful tool for developing lyophilized circRNA vaccines with targeted stability.

Altering vaccine pharmacokinetics by extending the translation of mRNA antigen has become an exciting tool to enhance antibody response (47). CircRNA vaccines are a promising choice to improve the stability and immunogenicity of mRNA vaccines (2). Because of the lack of free ends, circRNA has increased stability both *in vitro* and *in vivo*, which enables prolonged expression of target genes (4). In this study, similar to other research, we found that the antigen production of circRNA was higher (7). A promising avenue in rational vaccine design for modulating GCs is the sustained delivery of antigen, which can more mimic natural infection (48). Sustained antigen availability during GC reactions has been shown to increase antibody production by approximately 10-fold (2, 49). These published studies are consistent with our data, where the circRNA-G-induced IgG titer increased approximately 396-fold and the nAb titer increased approximately 9.2-fold compared to mRNA-G. However, the duration of the antibody response is a complex phenomenon that can vary depending on the specific antigen and requires long-term data for a comprehensive understanding.

The targeted delivery of circRNA to desired organs remains a great challenge for *in vivo* applications of circRNA technology (50). The current LNP mRNA is unable to control cell specificity and can be taken up by almost any cell type near or far from the site of injection (51). The SARS-CoV-2 mRNA vaccine (BNT162b2) administered by i.m. injection was distributed mainly in the liver and injected site, leading to reversible hepatic damage in animals (52). Therefore, exploring a lymphoid organ-specific circRNA vaccine could be a promising strategy for developing next-generation circRNA vaccines. Similar to persistent antigen expression of circRNA, targeted lymph node delivery effectively increases antigen availability to GCs (28). CircRNAs were found in our study and other studies to be effective in enhancing and prolonging the immune response and achieving better durable immunity than mRNA vaccines (2). Notably, LNP-circRNA-G was still affected by viral infection within a short time after vaccination, although it achieved 100% mouse survival, similar to mLNP-circRNA-G. This slight effect seems to portend concern about the long-term protection potential of the LNP-circRNA-G vaccine. In the trial, a single dose of mLNP-circRNA-G vaccine was able to elicit a durable antibody response and protection within 6 months. Of course, determining the duration of this immune response requires longer monitoring data. We demonstrated that a single immunization with mLNP-circRNA-G targeting LNs, but not with the LNP-circRNA-G vaccine, elicited potent GC B and Tfh cell responses as well as LLPCs and MBCs. In the past 3 years, on the basis of mRNA vaccine technology, many studies have been devoted to improving the immune effect of SARS-CoV-2 vaccines (53). By constructing mLNP-circRNA-RBD vaccines, we observed that this strategy significantly improved the antibody response against SARS-CoV-2. This further shows that targeting circRNA to LNs can improve vaccine response.

We highlight the importance of spatial and temporal cues in innate immune cell activation, the B-cell response, and the generation of a potent and durable adaptive immune response. This improvement in the immune response is based on the targeting modification of LNPs, which is also true in research on the targeted delivery of other mRNA vaccines (28, 54). To date, lyophilization technology has been widely used to improve the stability of mRNA vaccines, but lyophilization research basically focuses on exploring the influence of lyophilization on mRNA and LNPs, and it is rare to report whether lyophilization affects the targeting modification of the LNP surface (16, 17, 19). However, for LNP-mRNA vaccines that rely on targeting modification to improve vaccine efficacy, the stability of targeting modification on the LNP surface is very important, especially for lyophilized LNP-mRNA vaccines that need long-term preservation. In this study, we evaluated the effect of lyophilization on the targeted stability and immune efficacy of the mLNP-circRNA vaccine. The results showed that LNPs were prepared by a one-step method after PEG lipids were directly modified with mannose, and the lyophilization process had no significant effect on the targeting modification of mLNPs. Even after 24 weeks of storage at 4℃, the targeting and immunogenicity of mLNP-circRNA vaccine did not change significantly. The effects of lyophilization on the targeted stability of LNP-mRNA vaccines with different targeting modifications need to be studied in detail. Lyophilization is a promising choice until there is a major breakthrough in the stability of mRNA and LNPs. Therefore, for targeting modified LNP-mRNA vaccines, the influence of lyophilization on the stability of targeting modification needs to be studied.

T cells, although unable to prevent the virus from entering host cells following infection, play a crucial role in clearing rabies virus within mice (55). It has been reported that the RABV-G mRNA vaccine effectively induces RABV-G-specific T cells and, when compared to Rabipur, the mRNA vaccine leads to better induction of CD4^+^ T cells (38, 56). Therefore, further research is needed to explore the role of cellular immunity induced by the mLNP-circRNA-G vaccine in rabies vaccine protection. In summary, in this study, we focused on the future directions of mRNA vaccines, circRNA vaccines, targeted delivery and lyophilization, and analyzed the influence of lyophilization on the stability of LNP targeting modification and immunogenicity. We prepared a mLNP-circRNA vaccine targeting LNs, which retained long-term LN-targeting stability and immunogenicity after lyophilization. When mLNP-circRNA was subjected to an advanced lyophilization process to make it more thermostable and accessible, it entered the ranks of advanced and highly effective vaccines, solving the storage and transportation issues that hinder the use of existing LNP-mRNA vaccines. The development of new vaccines will particularly benefit from the design of generalizable platforms that are amenable to a broad array of antigens.

## MATERIALS AND METHODS

### Cells, viruses, reagents, and animals

HEK-293T, DC2.4 cells, and BSR cell lines were maintained in our laboratory. These cell lines were cultured in Dulbecco’ s modified Eagle medium (DMEM) (Merck, Cat. no. D5546) with 10% fetal bovine serum (FBS) (Gibco, Cat. no. 16000-044), supplemented with 1% penicillin–streptomycin (ThermoFisher Scientific, Cat. no. 15070063) in a 5% CO_2_ incubator at 37°C. The RABV virulent strain CVS-24 (challenge virus standard strain 24) was maintained in our laboratory as previously described (32, 57). CVS-24 is a mouse-adapted laboratory strain and propagated in the brains of newborn mice. The RABV challenge model based on the mouse adapted strain CVS-24 has been characterized in detail (32, 57). Antibodies directly labeled with fluorescein for flow cytometric analyses were purchased from Biolegend (CA, USA). PE anti-mouse CD95 antibody (Cat. no. 554295), 647 anti-mouse GL7 antibody (Cat. no. 144606), and FITC anti-mouse CD45R/B220 antibody (RRID:AB_312991) were used as markers of GC B cells in LNs; PE anti-mouse CD279 (PD1) antibody (Cat. no. 135206), APC anti-mouse CD185 (CXCR5) antibody (RRID:AB_2561970), and FITC anti-mouse CD4 antibody (RRID:AB_312713) were used as markers of Tfh cells in inguinal LNs; APC anti-mouse CD138 (Syndecan-1) antibody (RRID:AB_10962911) and FITC anti-mouse CD45R/B220 antibody (RRID:AB_312991) were used to analyze the number of PCs in BMs. HRP-labeled goat anti-mouse IgG (RRID:AB_2904507), IgG2b (RRID:AB_10695945), IgG2a (RRID:AB_10680049), and IgG1 (RRID:AB_10695944) for ELISA. Anti-G protein monoclonal antibody and anti-P protein monoclonal antibody were prepared in our laboratory as previously described (32). The RABV G protein was prepared as previously described (58). The SARS-CoV-2 spike (S) protein RBD (Cat. no. CG201-00) was purchased from Vazyme Biotech Co. Ltd. (Nanjing, China). Commercial inactivated rabies vaccine (ITV) was made by the Intervert International B.V. (Boxmeer, Netherlands). PEG-lipid (Cat. no. 06020112402), ionizable cationic lipid (SM102) (Cat. no. 06040008800), phosphatidylcholine (DSPC) (Cat. no. 06030001100), and cholesterol (Cat. no. 06040010300) were purchased from SINOPEG Biotechnology Co., Ltd. (Xiamen, China). ICR and C57BL/6 female mice (6- to 8-week-old) were purchased from Hunan SJA Laboratory Animal Co., Ltd. (Changsha, China). Mice were housed in the animal facility at Huazhong Agricultural University.

### DSPE-PEG2k-mannose synthesis

DSPE-PEG2k-NHS (500 mg) was weighed and dissolved in 3 mL of DMSO. Mannose (1.1 eq.) and triethylamine (2.0 eq.) were added to completely dissolve the solution. The solution was reacted for 2 h at 40°C. The reaction solution was transferred to a dialysis bag with a molecular weight cutoff of 1,000 Da. The solution was dialyzed in pure water for 24 h. The dialysate was collected and freeze-dried to obtain DSPE-PEG2k-mannose.

### mRNA production

mRNA was produced using T7 RNA polymerase on linearized plasmids encoding codon-optimized G protein of the RABV vaccine strain SAD (GenBank: EF206718.1). mRNA was transcribed to contain a 100 nucleotide-long poly(A) tail, with 100% of UTP substituted with 1-methylpseudo UTP to produce m1Ψ-modified mRNA. The m7G (5′) ppp (5′) G RNA Cap Structure Analog was used for cotranscriptional capping of mRNAs. Purified mRNA was analyzed by agarose gel electrophoresis and stored at −80°C until further use. The sequences of mRNA-G are provided in Table S1.

### CircRNA production

CircRNAs were produced according to previous reports (7). In brief, the circRNA precursors were synthesized via IVT from the linearized circRNA plasmid templates with the T7 High Yield RNA Transcription Kit (Novoprotein Scientific Inc., China). After IVT, the RNA products were treated with DNase I for 35 min to digest the DNA templates. After DNase I digestion, GTP was added to the reaction at a final concentration of 2 mM, and then the reactions were incubated at 55°C for 20 min to catalyze the cyclization of circRNAs. Then, the RNA was column purified with RNA Clean Beads (Vazyme Biotech Co. Ltd., China). Then, the column-purified RNA was heated at 65°C for 5 min and cooled on ice. The reactions were treated with RNase R at 37°C for 30 min to further enrich the circRNAs. The RNase R-treated RNA was column purified. The sequences of circRNA-G and circRNA-RBD produced via Group I introns are provided in Tables S2 and S3.

### Preparation of mLNP-circRNA-G vaccines

RABV G-encoded circRNA (circRNA-G) was encapsulated in mLNPs using a self-assembly process in which an aqueous solution of mRNA at pH 4.0 was rapidly mixed with a solution of lipids dissolved in ethanol. The LNPs used in this study contained an ionizable cationic lipid, phosphatidylcholine, cholesterol, and DSPE-PEG2k-mannose at a ratio of 50:10:38.5:1.5 mol/mol and were encapsulated at an mRNA to lipid ratio of approximately 0.05 (wt/wt). mLNP-circRNA-G was stored at 4°C at an RNA concentration of approximately 0.1 mg/mL.

### Nanoparticle characterization

Measurements of particle size (DLS) and potential were performed using a Zetasizer Nano ZS instrument (Malvern Instrument Co., Ltd.). Nanoparticles at appropriate concentrations were added to l-cm test dishes and transferred to a dynamic light scattering instrument to measure particle size and surface electrical properties. Transmission electron microscopy (TEM) was performed after the dilution of LPP-mRNA nanoparticles loaded with mRNA, with 10 µL aliquots placed onto copper mesh and left to stand for 10 min. Excess liquid was removed with filter paper, and the morphology of LPP-mRNA nanoparticles was observed using a Talos L120C microscope (Thermo Fisher) under an infrared lamp.

### Lyophilization process

The mLNP-circRNA solution was added to the cryoprotectant and placed in a penicillin bottle, and lyophilization was performed in a glass chamber of a freeze dryer (ALPHA 2–4 LD plus). The sample was first frozen at −45°C for 2 h to become solid, this being followed by a primary dry cycle at −25°C for 45 h, and finishing with secondary drying at 25°C for 4 h. The obtained powder was collected, characterized, and stored at 4°C for further use.

### Endosomal escape experiments

DC2.4 cells were inoculated into confocal dishes (1 × 10^5^ cells) overnight to study the endosomal escape abilities of mLNP-circRNA-G. The cyanine3 (Cy3) fluorescent dye-labeled (red) mLNP-circRNA-G was cocultured with adherent DC2.4 cells, including removal of the solution at various specified times (2, 4, and 6 h), and the cells were washed 3 times with 1.5 mL of preheated PBS to remove free dead cells and unbound mLNP-circRNA-G. Then, LysoTracker DND-26 (green) was added to confocal dishes to mark the lysosomes of cells. After treatment, the cells were washed 3 times with preheated PBS and fixed in 1.5 mL of 4% paraformaldehyde for 15 min at room temperature. Finally, 1 × intracellular staining permeabilization wash buffer containing 1% DAPI (blue) was applied to mark the nuclei of the cells. Ten minutes later, the cells were washed three times with PBS again. The subcellular distribution of variation with time was monitored using confocal laser scanning microscopy (CLSM, Olympus, FV-300, IX71) to verify the endosomal escape of mLNP-circRNA-G nanoparticles.

### Mouse vaccination and serum collection

ICR mice (6- to 8-week-old) were randomly allocated to the indicated groups. Mice received a single-dose immunization of 2 µg of LNP-mRNA-G, LNP-circRNA-G, and mLNP-circRNA-G. As a positive control, mice were administered a 0.1 dose of inactivated vaccine (ITV), a commercial rabies vaccine containing inactivated RABV. Sterile PBS was used as a control. For mouse vaccination, ICR mice received intramuscular inoculation using a 1-mL sterile syringe. The sera of immunized mice were collected at specified times to detect the RABV-specific IgG endpoint GMTs and neutralizing antibodies. Each mouse was immunized with 5 µg of mLNP-circRNA-RBD, LNP-circRNA-RBD, or LyomLNP-circRNA-RBD as a booster at week 4.

### Enzyme-linked immunosorbent assay (ELISA)

All immunized mouse serum samples were heat-inactivated at 56°C for 30 min before use. The IgG antibody endpoint titer was measured by ELISA. Briefly, ELISA plates were coated overnight at 4°C with 6 ng/µL RABV G protein or 2 ng/µL SARS-CoV-2 RBD protein diluted coating buffer. The plates were blocked with the blocking buffer for 1  h and washed and cultured with diluted mouse sera for 2  h. Then, after 3 washes with wash buffer, horseradish peroxidase HRP-conjugated rabbit anti-mouse IgG diluted in 1% BSA at a 1:10,000 ratio was added to the plates and incubated at 37°C for 60 min. Then, the plates were washed 3 times with wash buffer and added to TMB substrates (100 µL/well) followed by incubation for 15–20 min. After incubation, 50 µL of 2 M H_2_SO_4_ was added. Optical densities were recorded at 450 nm using a SpectraMax 190 spectrophotometer (Molecular Devices, CA, USA). The IgG endpoint titer was defined as the dilution fold.

### RABV nAb measurement

As previously described (59), virus nAb titers were measured using fluorescent-antibody virus neutralization (FAVN) assays. Briefly, mouse serum was separated and inactivated for 30 min at 56°C. Then, 100 µL of DMEM was added to 96-well plates, and 50 µL of test serum or standard serum was added to the first column and serially diluted 3-fold. Each sample was added to four adjacent wells. A RABV (CVS-11) suspension was added to each well. The plates were then incubated at 37°C for 1 h. After incubation, 2 × 10^4^ BSR cells were added to each well, and the solutions were incubated at 37°C for 72 h. Samples were then fixed with 80% ice-cold acetone for 30 min and stained with FITC-conjugated antibodies against the RABV N protein. Fluorescence was observed under an Olympus IX51 fluorescence microscope (Olympus, Tokyo, Japan). Fluorescence values were compared with reference serum values (obtained from the National Institute for Biological Standards and Control, Hertfordshire, UK). The results were normalized and quantified in international units per mL (IU/mL).

### Pseudovirus neutralization assay

SARS-CoV-2 neutralizing antibody titers were tested as previously reported (60). Briefly, the pseudovirus was diluted with DMEM and incubated with 2-fold serially diluted mouse sera for 1 h at 37°C. Then, HEK293T-hACE2 cells were added and incubated for 24 h. Luciferase (Luc) activity was measured using a microplate reader. The neutralization endpoint NT_50_ is defined as the serum fold dilution required to inhibit 50% of luciferase activity.

### Flow cytometry

Tfh cells, GC B cells, and LLPCs isolated from draining LNs or BMs were analyzed by flow cytometry as previously described (61). LNs and BMs were collected at 7 or 14 dpi. LNs and BMs were harvested, and single-cell suspensions were obtained by passage of the LNs through a 70-µm filter (BD Biosciences). Red blood cells were removed using lysis buffer (Cat. no. 555899, BD Biosciences Inc., Franklin Lakes, NJ, USA). Cells were washed with PBS and labeled with fluorescence-conjugated antibodies for 30 min at 4°C. After incubation for 30 min at 4°C, the cells were washed twice with PBS containing 0.2% (wt/vol) BSA. Finally, stained cells were analyzed using a BD FACSVerse system. Data were analyzed using FlowJo software (TreeStar, CA, USA).

### ELISpot assay

ELISpot assays were conducted to analyze RABV-specific ASCs according to the manufacturer’s instructions (Millipore, MA, USA). The plates were generally coated with antigens (500 ng/well purified RABV-G) overnight at 4°CC. Then, the plates were washed and blocked for the ELISpot assays. Drained LNs were collected at 7 or 14 dpi and processed into single-cell suspensions. The next day, the coated plates were washed 3 times with PBS-Tween (PBST; 0.5% tween-80, wt/vol) and blocked with RPMI 1640 supplemented with 10% FBS for 2 h at 37°C. Cell suspensions were transferred to blocked ELISpot plates and incubated for 24 h. The assay was conducted with biotin-conjugated mouse IgG antibody (Bethyl Laboratories, TX, USA), streptavidin-alkaline phosphatase (Mabtech, Stockholm, Sweden), and BCIP/NBT-plus (Mabtech, Stockholm, Sweden). Then, plates were scanned and analyzed using a Mabtech IRIS FluoroSpot/ELISpot reader and RAWspot technology for multiplexing at the single-cell level.

### Mouse challenge experiments

A mouse model of rabies using the virulent RABV strain CVS-24 has previously been characterized (61). An intracranial challenge with 30 µL of 50 LD_50_ (median lethal doses) CVS-24 was performed at 3 weeks or 6 months post immunization. The body weights and mortality of the mice were recorded daily. A second group of mice was similarly treated and euthanized at the indicated times, and the brains were harvested for hematoxylin and eosin (H&E) staining or IHC/IHC-F analysis.

### Histopathology assay

For histopathology, brain tissue samples were collected from euthanized mice. The collected tissues were fixed in a 4% (vol/vol) paraformaldehyde solution for 48 h. Subsequently, 3–4 µm thick paraffin sections were prepared and stained using hematoxylin and eosin (H&E) to identify tissue histopathological changes. Observation was carried out using an optical microscope, and analysis was conducted based on three independent replicates.

### Immunofluorescence staining of brain tissues

The expression of the P protein in brain tissues was determined by immunofluorescence staining, serving as a marker for RABV within the brain tissue. Brain tissue paraffin sections were deparaffinized in xylene, rehydrated through a series of ethanol/water, and then incubated in 3% H_2_O_2_ at room temperature. Subsequently, the sections were placed in a 10 mM sodium citrate buffer (pH 6) and subjected to antigen retrieval at 95°C for 1 h. A 30 min blocking step with saturated BSA followed. The primary antibody against RABV P protein was incubated for 2.5 h at 37°C in a humidified chamber, followed by detection using a secondary antibody conjugated with Cy3 (Servicebio). Images were captured using a LEICA Versa 200 microscope and processed using Caseviewer C. V 2.4 and Image-Pro Plus 6.0 software.

### Safety and stability profiles of mLNP-circRNA-G

The cytotoxicity of mLNP-circRNA-G was measured by the MTT assay. An acute toxicity test was used to evaluate the preliminary safety of mLNP-circRNA-G. Female ICR mice were treated with mLNP-circRNA-G containing 30 µg of circRNA-G by intramuscular administration. After 24 h, the serum samples were obtained through centrifugation. Related biochemical indicators, including ALT, AST, total protein, and urea nitrogen, were detected by an automatic hematological biochemical analyzer (Hitachi High-Technologies Corp., Minatoku, Tokyo, Japan). The organ tissues of the mice were collected for H&E staining. All slide images were taken with an Olympus-BX 43 fluorescence microscope (Olympus Corp., Tokyo, Japan).

mLNP-circRNA-G was stored at 4°C for 0, 7, 14, and 28 days to study its stability by observing the changes in particle size, zeta potential, mRNA encapsulation efficiency, IgG titer and nAb titer. Mice (*n* = 3) were injected once intramuscularly with mLNP-circRNA-G and circRNA-G on day 0. Serum samples were collected on day 14 and analyzed by ELISA.

### Statistical analyses

Statistics were analyzed using GraphPad Prism software 9.0 (GraphPad Software, Inc., CA). Survival statistical analysis was performed using a log-rank (Mantel‒Cox) test. For other data, significant differences between groups were analyzed by using one-way ANOVA followed by post hoc tests. The following notations were used to indicate significant differences between groups: *P < 0.05; **P < 0.01; ***P < 0.001; ****P < 0.0001; ns, no significant difference.

## Data Availability

The main data supporting the findings of this study are available within the paper and its supplementary materials. The data not shown are available from the corresponding author (L.Z.). All unique reagents generated in this study, such as circRNA, mRNA and lipid, are available upon reasonable request under a completed Material Transfer Agreement.

## SUPPLEMENTAL MATERIAL

Supplemental Material mbio.01775-23-s0001.pdf Supplemental figures and table.

ASM does not own the copyrights to Supplemental Material that may be linked to, or accessed through, an article. The authors have granted ASM a non-exclusive, world-wide license to publish the Supplemental Material files. Please contact the corresponding author directly for reuse.
