# Supplementary material for: Circular RNA vaccines with long-term lymph node-targeting delivery stability after lyophilization induce potent and persistent immune responses
Source: mBio. 2023 Dec 11;15(1):e01775-23. doi: 10.1128/mbio.01775-23 (PMC10790773; doi:10.1128/mbio.01775-23)
Supplement: Supplemental Material — Supplemental figures and table. [file mbio.01775-23-s0001.pdf]

Supplemental Material

Titer:

Circular RNA vaccines with long-term lymph node-targeting delivery stability after lyophilization induce potent and persistent immune responses

Jiawu Wan[1,2,3#], Zongmei Wang[1,2,3#], Lingli Wang[1,2,3], Liqin Wu[1,2,3], Ming Zhou[1,2,3], Chengguang Zhang[1,2,3], Huanchun Chen[1,2,3], Zhen F. Fu[1,2,3], Ling Zhao[1,2,3]*

[1]National Key Laboratory of Agricultural Microbiology, Huazhong Agricultural University, Wuhan, China

[2] Hubei Hongshan Laboratory, Wuhan, China

[3] Key Laboratory of Preventive Veterinary Medicine of Hubei Province, College of Veterinary Medicine, Huazhong Agricultural University, Wuhan, China

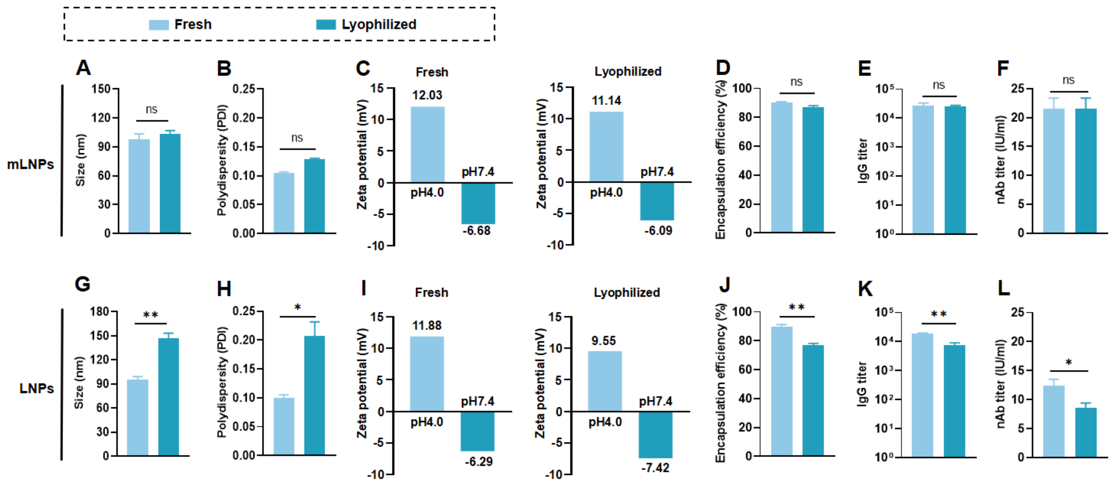

Figure S1. Mannose modification improved the stability of LNPs after lyophilization. Lyophilized LNP-circRNA-G and mLNP-circRNA-G were rehydrated for analysis of their physical properties. Mice were immunized once, and serum samples were collected on day 14 for antibody detection. (A-L) Particle size (A, G), PDI (B, H), zeta potential (C, I), mRNA encapsulation efficiency (D, J), IgG titer (E, K) and nAb titer (F, L).

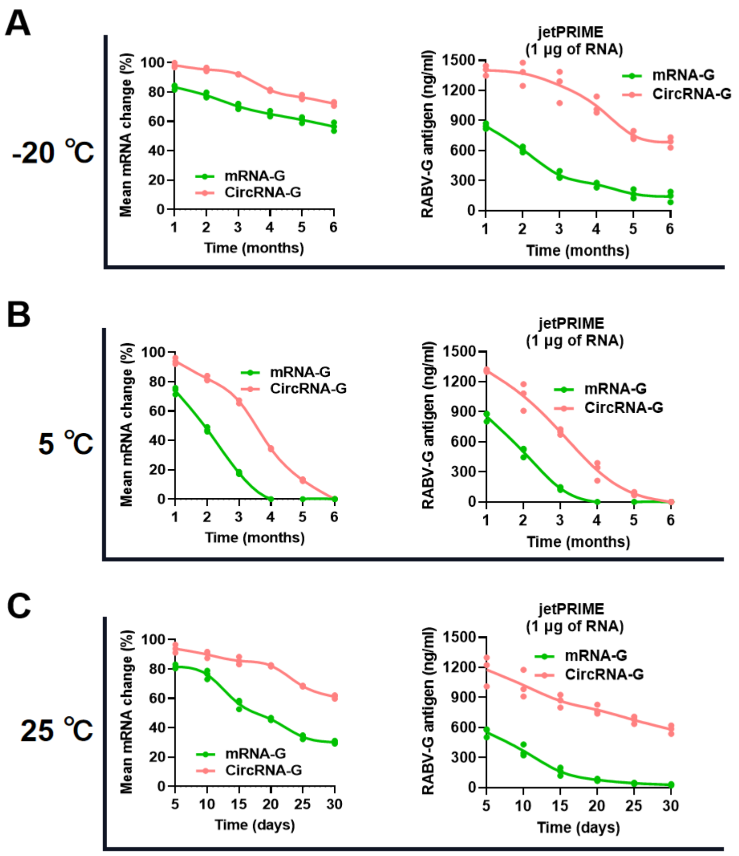

Figure S2. CircRNA-G vaccines are more stable than mRNA-G vaccines. (A-C) The degradation rate and antigen expression of circRNA-G and mRNA-G were stored at different temperatures.

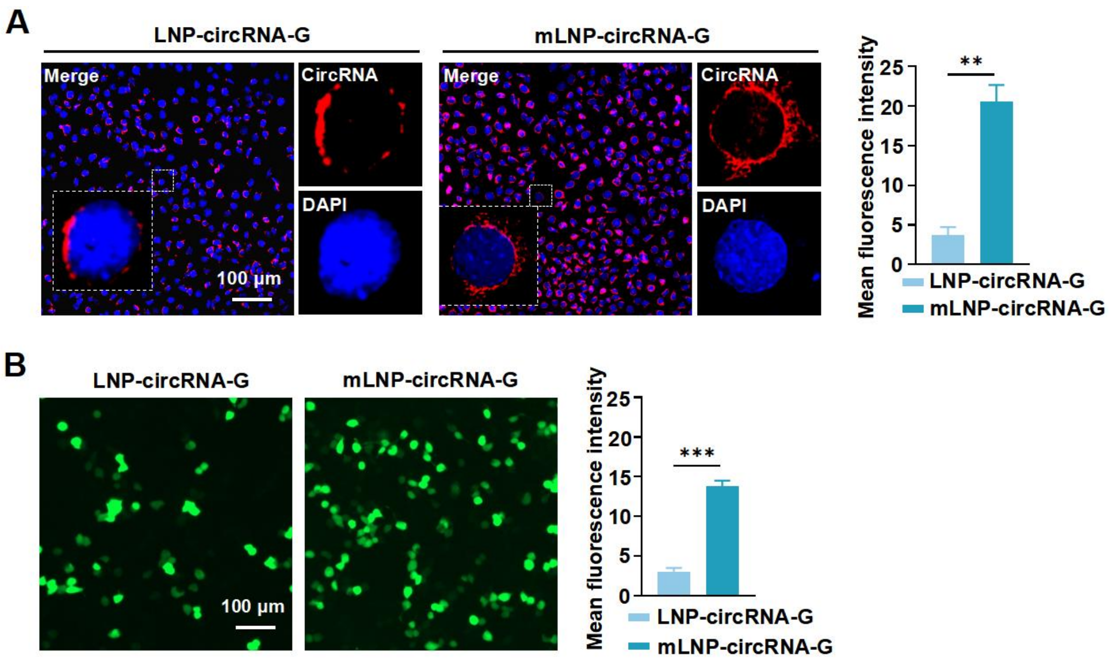

Figure S3. Cellular internalization of mLNP-circRNA-G nanoparticles. (A) Cellular uptake of mLNP-circRNA-G or LNP-circRNA-G in DC2.4 cells. Scale bar, 100 μm. (B) mLNP-circRNA-G or LNP-circRNA-G was transfected into DC2.4 cells. Cells were harvested after incubation for 24 h and detected using G protein monoclonal antibodies. G protein expression was visualized using inverted fluorescence microscopy.

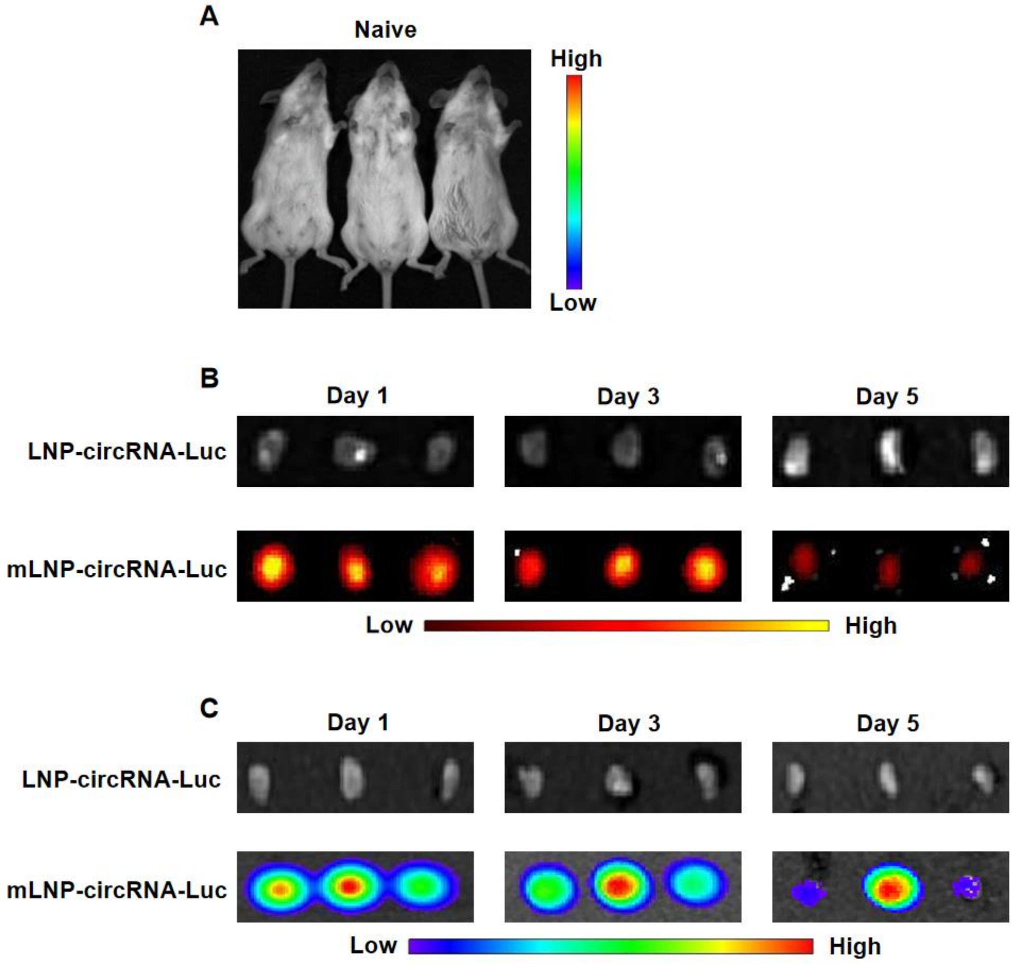

Figure S4. LN targeting. (A) Bioluminescence imaging of untreated mice, related to Figure 3B. Fluorescence (B) and bioluminescence (C) imaging of excised dLNs at 1, 3 and 5 dpi.

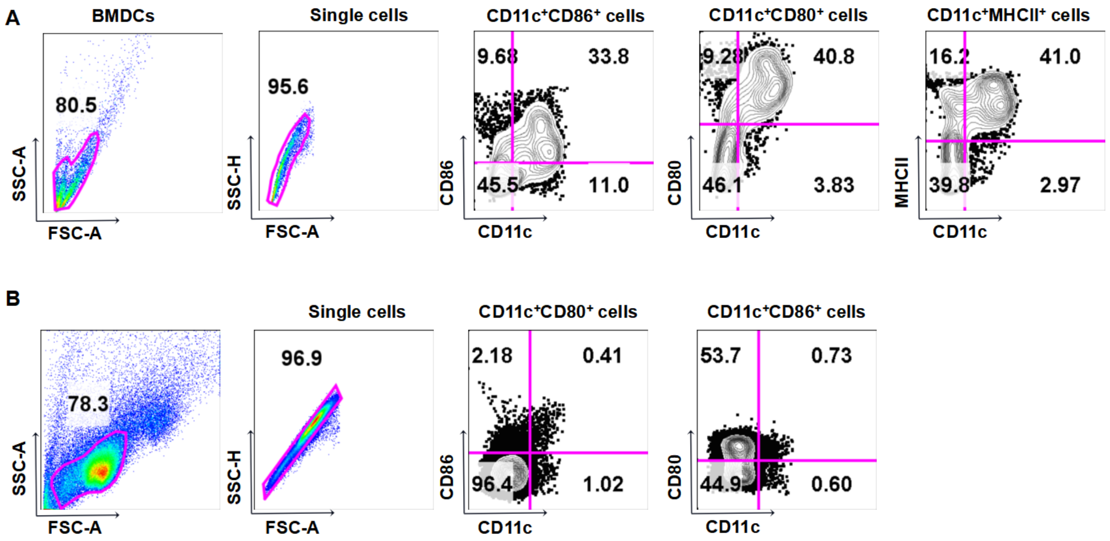

Figure S5. Gating strategy of intracellular staining for flow cytometry. (A) The gating strategy of mature BMDCs (CD11c$^+$CD86$^+$, CD11c$^+$CD80$^+$, or CD11c$^+$MHCII$^+$ cells). (B) The gating strategies of mature cDCs (CD11c$^+$CD80$^+$ and CD11c$^+$CD86$^+$ cells) in LNs.

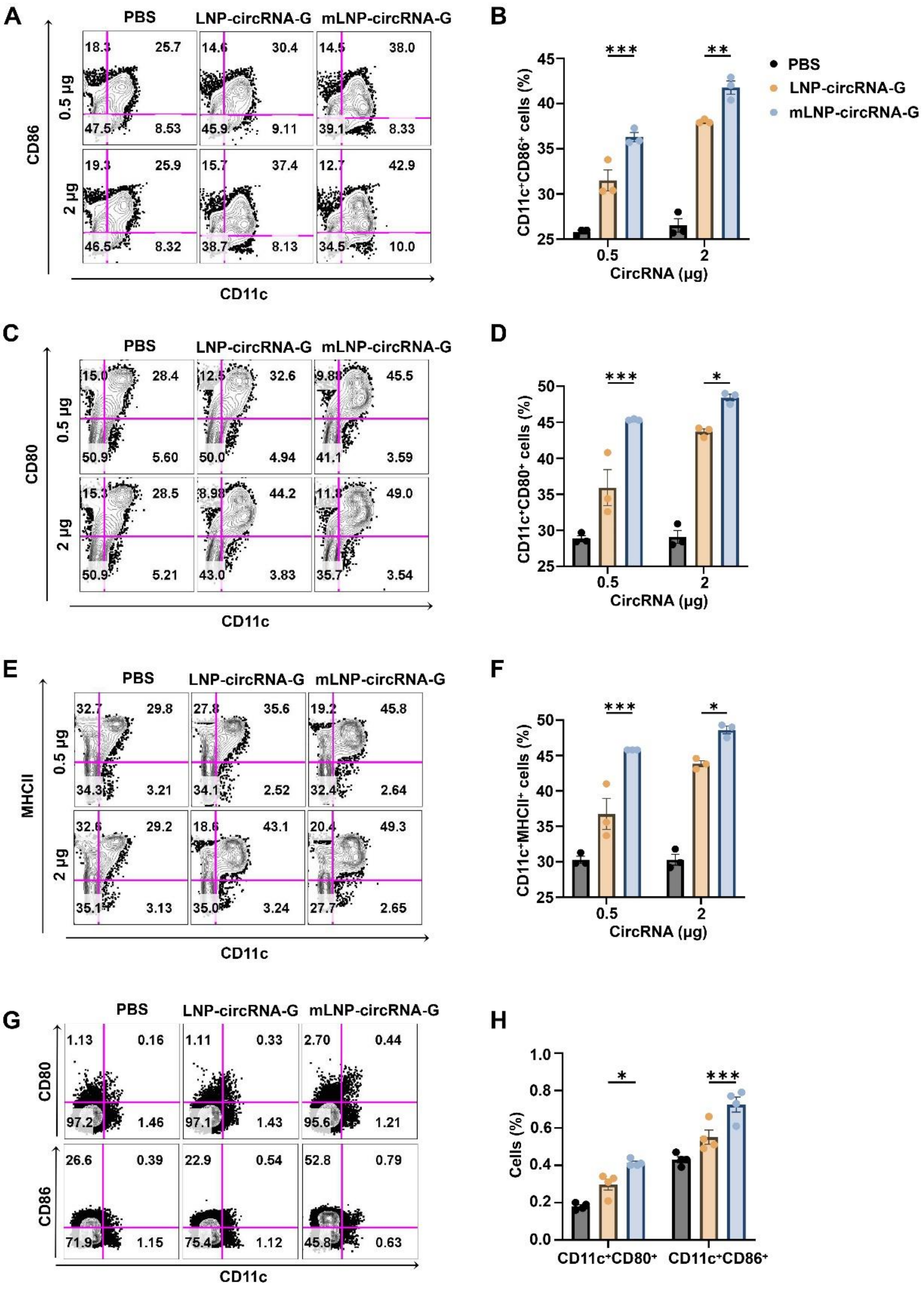

Figure S6. Effect of mLNP-circRNA-G on the maturation of DCs in vitro and in vivo. (A) Representative flow cytometric plots of CD11c+CD86+ cells. BMDCs were treated with 0.5 μg or 2 μg of mLNP-circRNA-G or LNP-circRNA-G or DMEM. At 24 h, the cells were harvested and subjected to flow cytometric analysis. (B) Statistical results of CD11c+CD86+ cells. (C) Representative flow cytometric plots of CD11c+CD80+ cells. (D) Statistical results of CD11c+CD80+ cells. (E) Representative flow cytometric plots of CD11c+MHCII+ cells. (F) Statistical results of CD11c+ MHCII+ cells. (G) Representative flow cytometric plots of CD11c+CD80+ DCs and CD11c+CD86+ DCs in LNs. (H) Statistical results of mature cDCs in LNs.

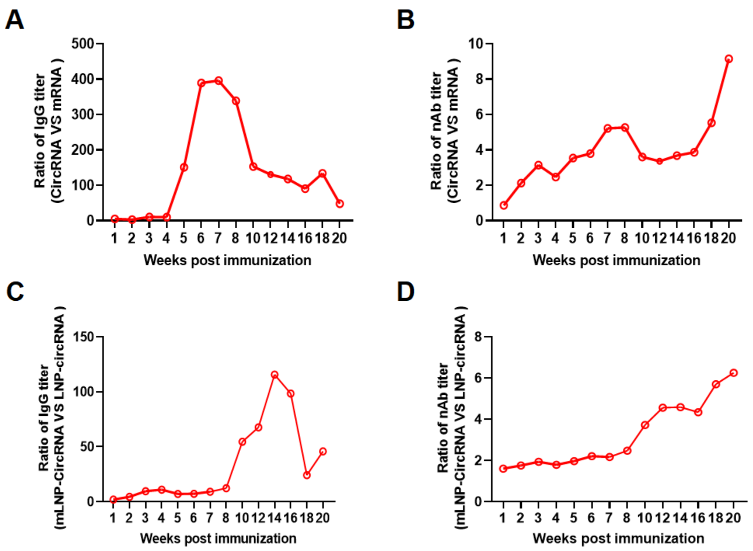

Figure S7. CircRNA-G and LN-targeting delivery immunization induces higher Ab titers. (A-B) The ratio of IgG and nAb titers induced by the LNP-circRNA-G vaccine to that induced by the LNP-mRNA-G vaccine. (C-D) The ratio of IgG and nAb titers induced by the mLNP-circRNA-G vaccine to that induced by the LNP-circRNA-G vaccine.

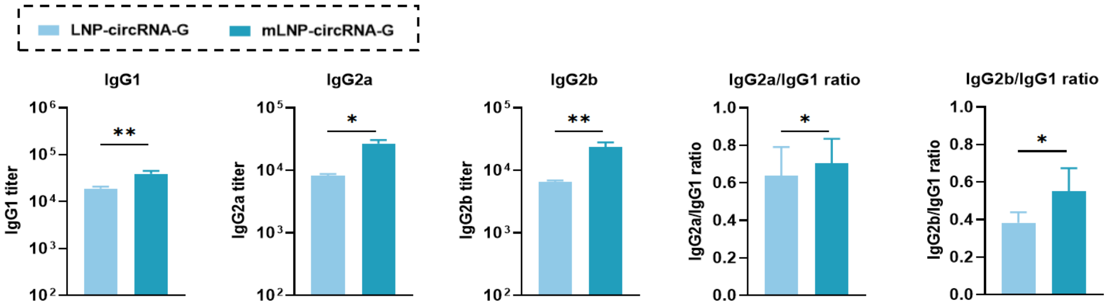

Figure S8. A balanced Th1/Th2 immune response was induced by mLNP-circRNA-G. ICR mice were immunized with a single injection of 2 μg of mLNP-circRNA-G or LNP-circRNA-G. Sera were collected week 4 post immunization and assessed by ELISA for RABV G-specific IgG1, IgG2a and IgG2b titers. Titer ratios of IgG2a to IgG1 and IgG2b to IgG1 were calculated. Data are means ± SEMs (n = 10).

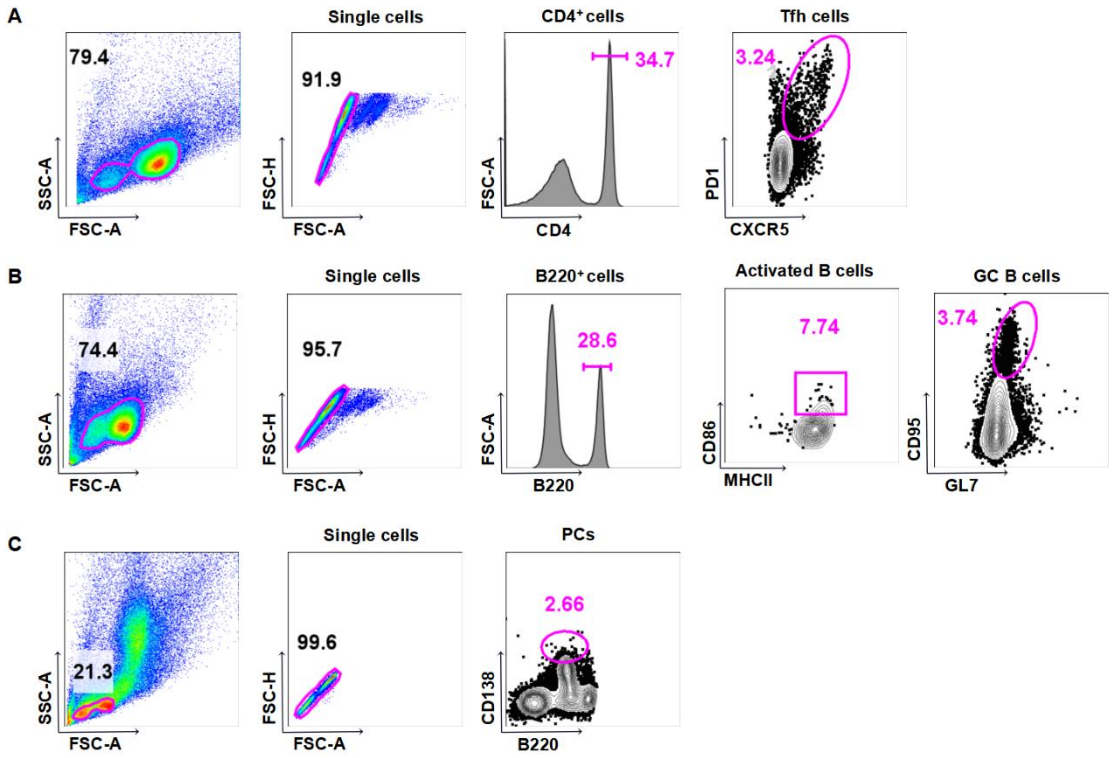

Figure S9. Gating strategy of staining for flow cytometry. (A) The gating strategy of Tfh ($CD4^+CXCR5^+PD1^+$) cells in LNs. (B) The gating strategies of activated B cells ($B220^+MHCII^+CD86^+$ cells) and GC B cells ($B220^+GL7^+$ $CD95^+$ cells) in LNs. (C) The gating strategy of LLPCs ($B220^{low}CD138^+$ cells) in BMs.

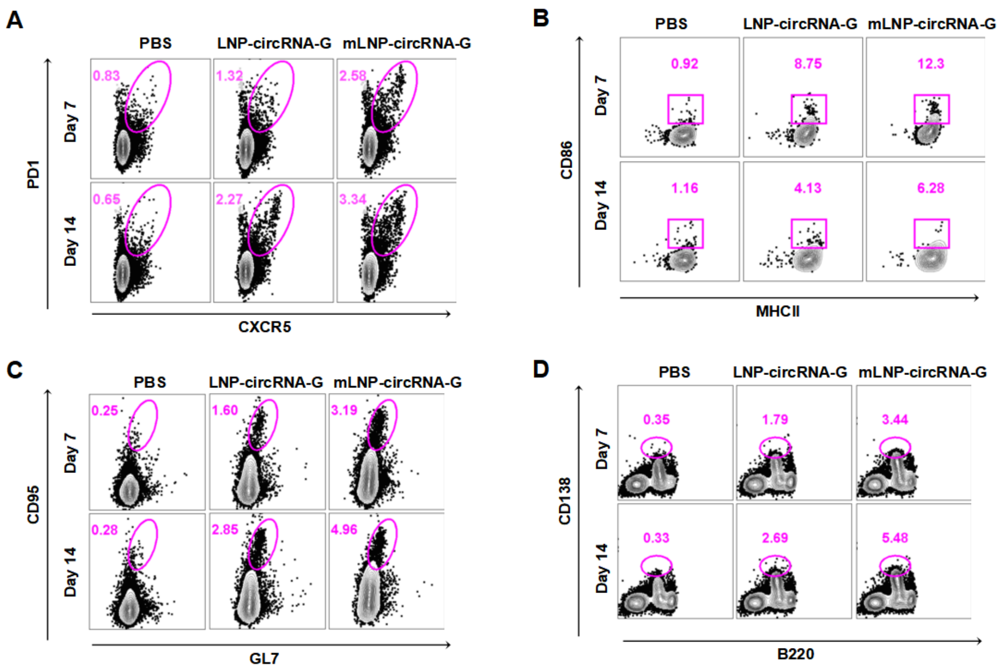

Figure S10. mLNP-circRNA-G facilitates the generation of Tfh cells, activated B cells, GC B cells, LLPCs and RABV-specific ASCs. (A) Representative flow cytometric plots of Tfh ($CD4^+CXCR5^+PD1^+$) cells. C57BL/6 mice were immunized with 2 μg of mLNP-circRNA-G, LNP-circRNA-G, or DMEM (n = 4). Draining LNs or BMs were collected at 7 dpi and 14 dpi, and cell suspensions were analyzed by flow cytometry. (B) Representative flow cytometric plots of activated B cells ($B220^+MHCII^+CD86^+$). (C) Representative flow cytometric plots of GC B cells ($B220^+GL7^+CD95^+$). (D) Representative flow cytometric plots of LLPCs ($B220^{low}$ $CD138^+$).

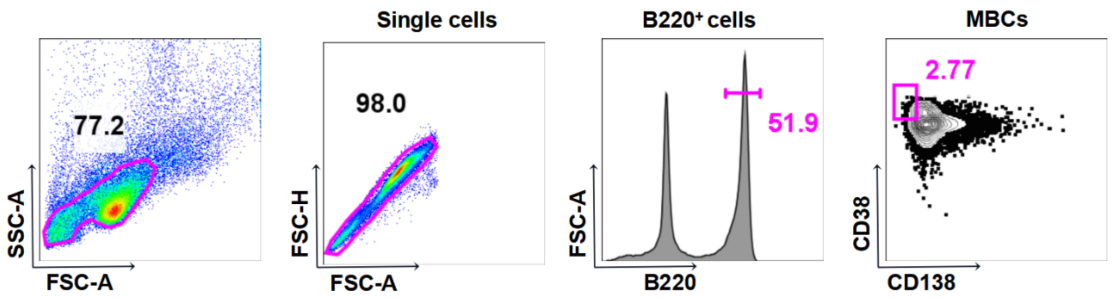

Figure S11. Gating strategy of staining for flow cytometry. The gating strategies of MBCs ($B220^{+}CD38^{+}CD138^{-}$ cells) in LNs.

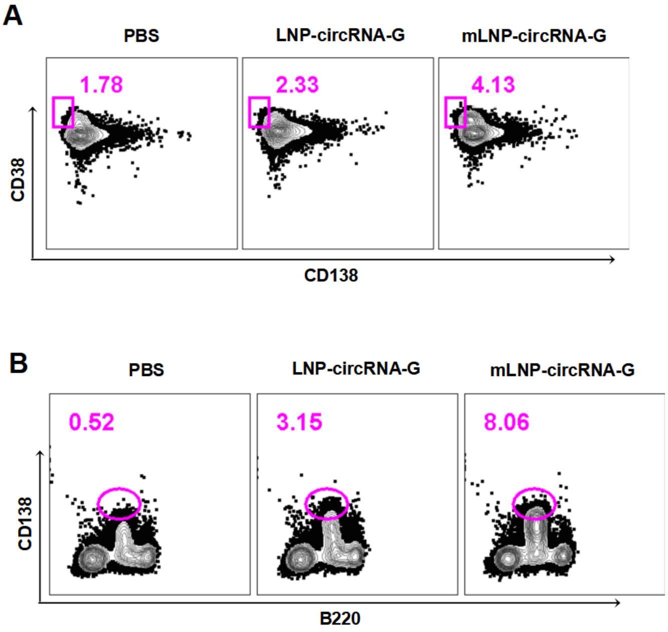

Figure S12. mLNP-circRNA-G induces MBC generation and secondary antibody responses. (A) Representative flow cytometric plots of MBCs ($B220^{+}CD38^{+}CD138^{-}$) in LNs. C57BL/6 mice (n = 4) were immunized with 2 μg of mLNP-circRNA-G, LNP-circRNA-G, or DMEM. At 100 dpi, single LN cells were stained with antibodies to analyze the MBCs by flow cytometry. (B) Representative flow cytometric plots of LLPCs ($B220^{low}$ $CD138^{+}$). Mice were boosted with 2 μg of mLNP-circRNA-G or LNP-circRNA-G at 100 days after primary immunization. At 14 days after the boost, LLPCs in BMs were evaluated by flow cytometry.

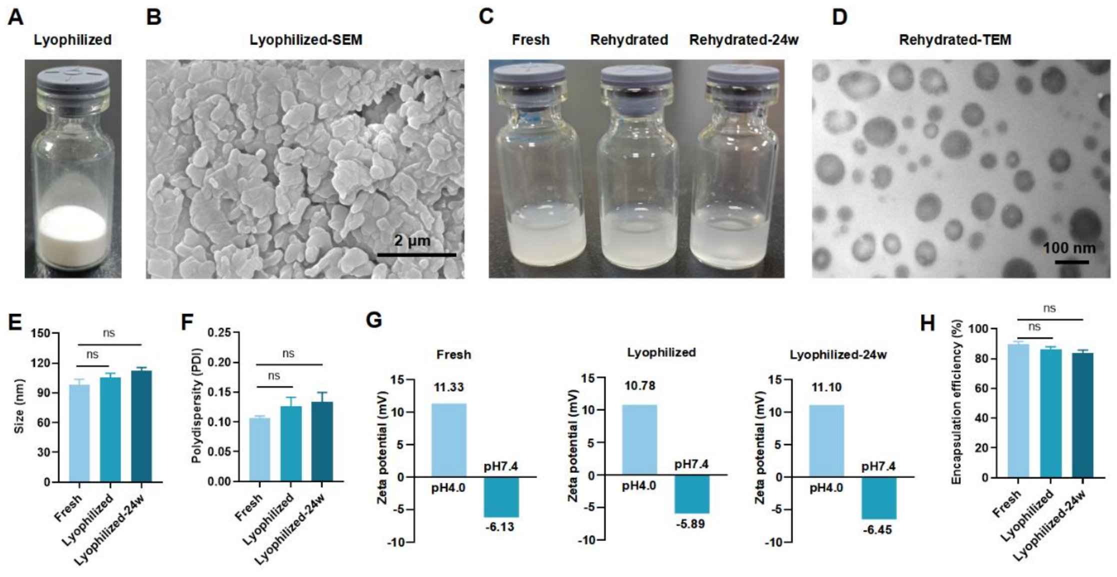

Figure S13. Lyophilized mLNP-circRNA-G exhibits long-term stability. (A) Image of lyophilized mLNP-circRNA-G. (B) SEM image of lyophilized mLNP-circRNA-G. (C) Image of fresh mLNP-circRNA-G, lyophilized mLNP-circRNA-G and the rehydrated solutions. (D) TEM image of rehydrated mLNP-circRNA-G. (E-H) Long-term stability of lyophilized mLNP-circRNA-G. Changes in the size (E), PDI (F), zeta potential (G) and encapsulation efficiency (H) of lyocircRNA-G mLNPs after storage at 4 ℃ for 24 weeks.

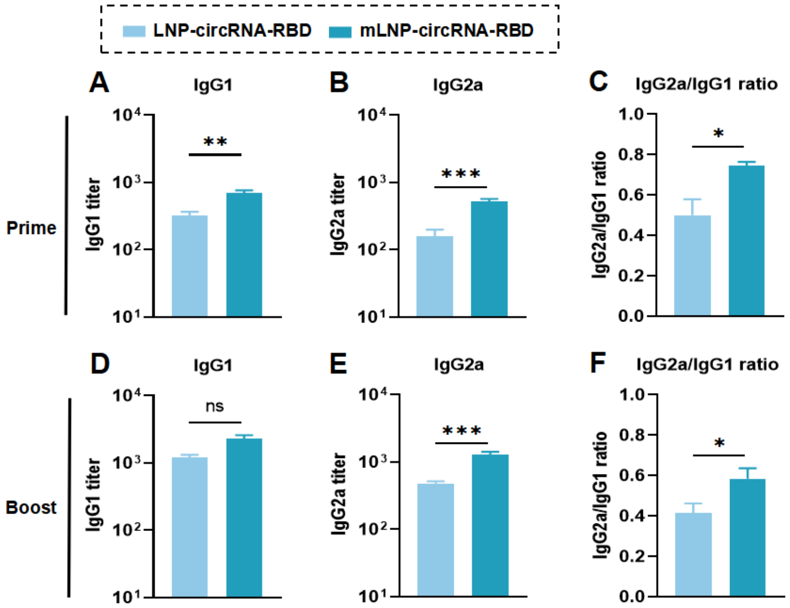

Figure S14. A balanced Th1/Th2 immune response is induced by mLNP-circRNA-RBD. ICR mice were immunized with a single injection of 5 µg of mLNP-circRNA-RBD or LNP-circRNA-RBD. Sera were collected at week 4 after primary and booster immunization and assessed by ELISA for RABV G-specific IgG1 and IgG2a titers. Titer ratios of IgG2a to IgG1 were calculated. Data are means ± SEMs (n = 5).

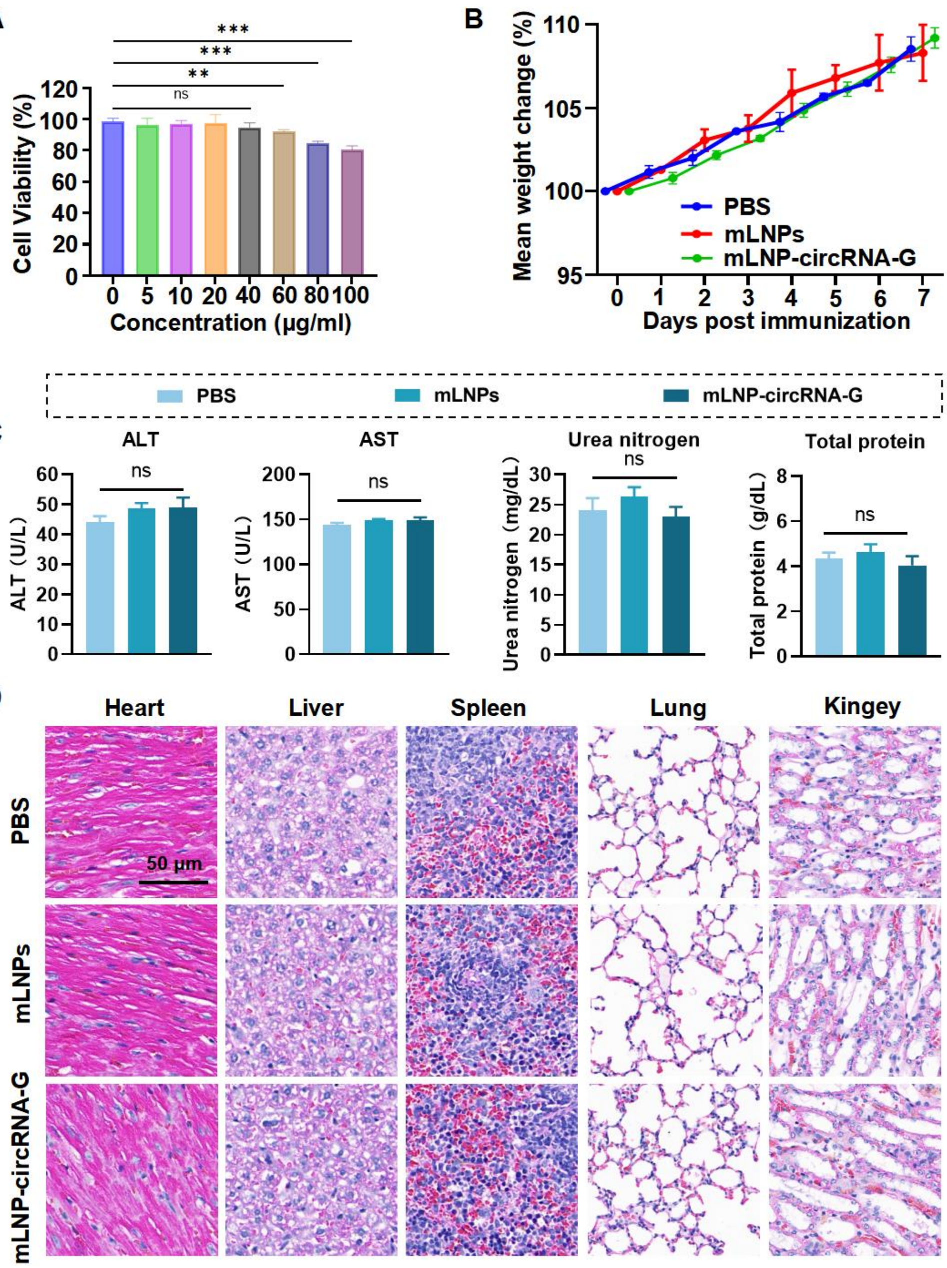

Figure S15. Safety evaluation of mLNP-circRNA-G *in vivo* and *in vitro*. (A) Cytotoxicity analysis of LPP-mRNA-G in HEK-293T cells by MTT assays. One-way ANOVA was used to evaluate intergroup differences. ns, no significant difference; Error bars represent SEM (n = 3). (B) ICR mice were injected intramuscularly with 30 μg of mLNPs or mLNP-circRNA-G for 24 h to evaluate the safety of mLNP-circRNA-G. Body weight changes in the mice were continuously monitored. Error bars represent SEM (n = 3). (C) Biochemical indicators of mLNPs and mLNP-circRNA-G-vaccinated mice were detected by an automatic hematological biochemical analyzer. (D) H&E analysis of major organs from mLNPs and mLNP-circRNA-G-vaccinated mice on day 2. Scale bars, 50 μm.

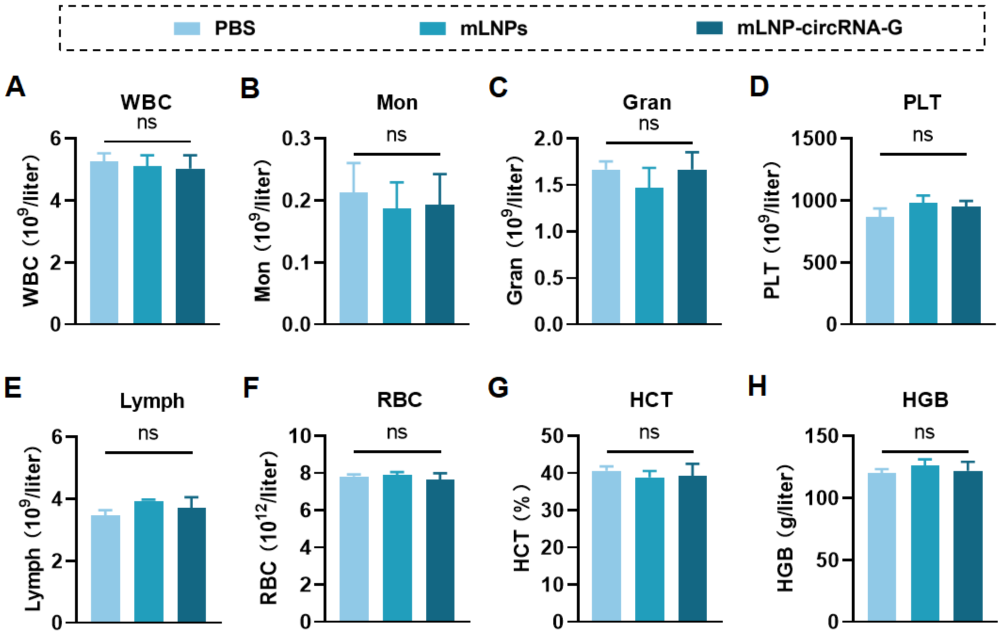

Figure S16. Biochemical indicators of mLNPs and mLNP-circRNA-G-vaccinated mice. (A-H) Biochemical indicators of mLNPs and mLNP-circRNA-G-vaccinated mice were detected by an automatic hematological biochemical analyzer.

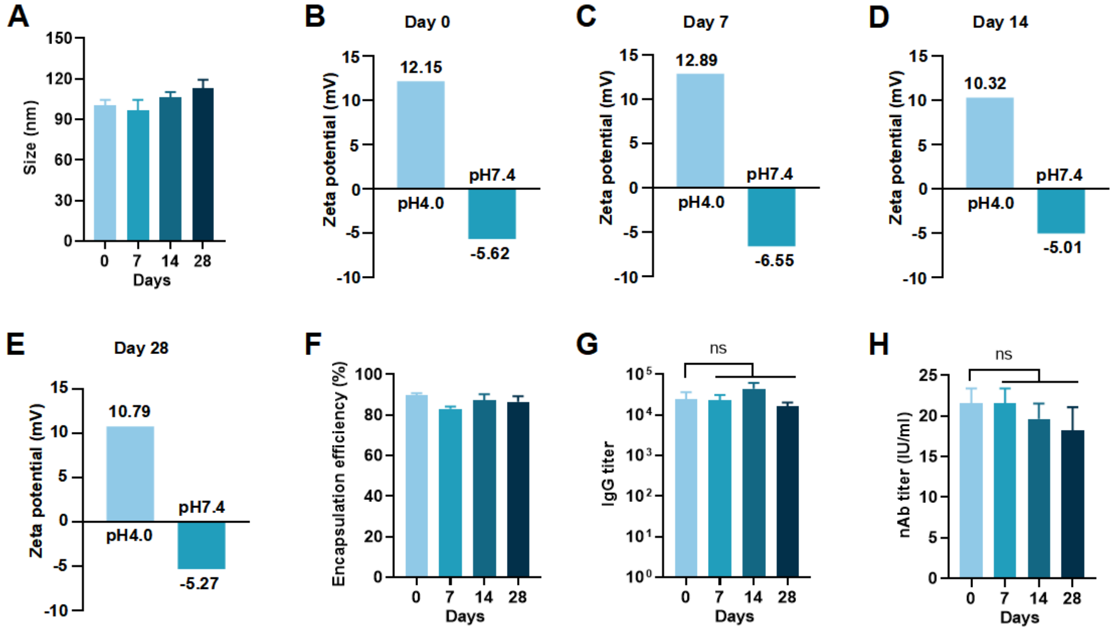

Figure S17. Stability evaluation of mLNP-circRNA-G. (A-H) Particle size (A), zeta potential (B-E), mRNA encapsulation efficiency (F), IgG titer (G) and nAb titer (H) (ICR mice were immunized once and serum samples were collected on day 14) were detected after mLNP-circRNA-G storage at 4 °C for 0, 7, 14 and 28 days.

Supplemental Table

Table S1. The sequences of the mRNA-G.

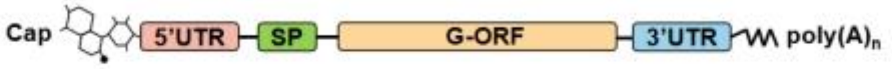

IVT template_mRNA-G

5'-
GGGAAATAAGAGAGAAAAGAAGAGTAAGAAGAAATATAAGACCCCGGCGCCGCCACCAA
GCTT**ATG**TTCGTGTTCCTGGTGCTGCTGCCCCTGGTGAGCAGCCAGTGCGTGGTTCCTCAG
GCTCTCCTGTTTGTACCCCTTCTGGTTTTTCCATTGTGTTTTGGGAAATTCCCTATTTACACG
ATACCAGACAAGCTTGGTCCCTGGAGTCCGATTGACATACATCACCTCAGCTGCCCAAACA
ATTTGGTAGTGGAGGACGAAGGATGCACCAACCTGTCAGGGTTCTCCTACATGGAACTTAA
AGTTGGATACATCTTAGCCATAAAAGTGAACGGGTTCACTTGCACAGGCGTTGTGACGGAG
GCTGAAACCTACACTAACTTCGTTGGTTATGTCACAACCACGTTCAAAAGAAAGCATTTCC
GCCCAACACCAGATGCATGTAGAGCCGCGTACAACTGGAAGATGGCCGGTGACCCCAGATA
TGAAGAGTCTCTACACAATCCGTACCCTGACTACCGCTGGCTTCGAACTGTAAAAACCACC
AAGGAGTCTCTCGTTATCATATCTCCAAGTGTGGCAGATTTGGACCCATATGACAGATCCCT
TCACTCGAGGGTCTTCCCTAGCGGGAAGTGCTCAGGAGTAGCGGTGTCTTCTACCTACTGC
TCCACTAACCACGATTACACCATTTGGATGCCCGAGAATCCGAGACTAGGGATGTCTTGTGA
CATTTTTACCAATAGTAGAGGGAAGAGAGCATCCAAAGGGAGTGAGACTTGCGGCTTTGTA
GATGAAAGAGGCCTATATAAGTCTTTAAAAGGAGCATGCAAACTCAAGTTATGTGGAGTTCT
AGGACTTAGACTTATGGATGGAACATGGGTCTCGATGCAAACATCAAATGAAACCAAATGG
TGCCCTCCCGATAAGTTGGTGAACCTGCACGACTTTCGCTCAGACGAAATTGAGCACCTTG
TTGTAGAGGAGTTGGTCAGGAAGAGAGAGGAGTGTCTGGATGCACTAGAGTCCATCATGA
CAACCAAGTCAGTGAGTTTCAGACGTCTCAGTCATTTAAGAAAACTTGTCCCTGGGTTTGG
AAAAGCATATACCATATTCAACAAGACCTTGATGGAAGCCGATGCTCACTACAAGTCAGTCA
GAACTTGGAATGAGATCCTCCCTTCAAAAGGGTGTTTAAGAGTTGGGGGGAGGTGTCATCC
TCATGTGAACGGGGTGTTTTTCAATGGTATAATATTAGGACCTGACGGCAATGTCTTAATCCC
AGAGATGCAATCATCCCTCCTCCAGCAACATATGGAGTTGTTGGAATCCTCGGTTATCCCCC
TTGTGCACCCCCTGGCAGACCCGTCTACCGTTTTCAAGGACGGTGACGAGGCTGAGGATTT
TGTTGAAGTTCACCTTCCCGATGTGCACAATCAGGTCTCAGGAGTTGACTTGGGTCTCCCG
AACTGGGGGAAGTATGTATTACTGAGTGCAGGGGCCCTGACTGCCTTGATGTTGATAATTTT
CCTGATGACATGTTGTAGAAGAGTCAATCGATCAGAACCTACGCAACACAATCTCAGAGGG
ACAGGGAGGGAGGTGTCAGTCACTCCCCAAAGCGGGAAGATCATATCTTCATGGGAATCAC
ACAAGAGTGGGGGTGAGACCAGACTG**TGATAATAG**GGTACCGCTGGAGCCTCGGTGGCCT
AGCTTCTTGCCCCTTGGGCCTCCCCCCAGCCCCTCCTCCCCTTCCTGCACCCGTACCCCCGT
GGTCTTTGAATAAAGTCTGAGTGGGCGGCA-3'

Table S2. The sequences of circRNA-G produced via group I intron.

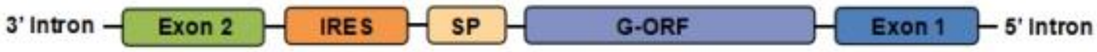

IVT template_circRNA-G

5'-

AACAATAGATGACTTACAACTAATCGGAAGGTGCAGAGACTCGACGGGAGCTACCCTAACG
TCAAGACGAGGGTAAAGAGAGAGTCCAATTCTCAAAGCCAATAGGCAGTAGCGAAAGCTG
CAAGAGAATGAAAATCCGTTGACCTTAAACGGTCGTGTGGGTTCAAGTCCCTCCACCCCCA
CGCCGGAAACGCAATAGCCGAAAAACAAAAAACAAAAAAAACAAAAAAAAAACCAAAA
AAACAAAACACATTAAAACAGCCTGTGGGTTGATCCCACCCACAGGCCCATTGGGCGCTAG
CACTCTGGTATCACGGTACCTTTGTGCGCCTGTTTTATACCCCCTCCCCCAACTGTAACTTAG
AAGTAACACACACCGATCAACAGTCAGCGTGGCACACCAGCCACGTTTTGATCAAGCACTT
CTGTTACCCCGGACTGAGTATCAATAGACTGCTCACGCGGTTGAAGGAGAAAGCGTTCGTT
ATCCGGCCAACTACTTCGAAAAACCTAGTAACACCGTGGAAGTTGCAGAGTGTTTCGCTCA
GCACTACCCCAGTGTAGATCAGGTCGATGAGTCACCGCATTCCCCACGGGCGACCGTGGCG
GTGGCTGCGTTGGCGGCCTGCCCATGGGGAAACCCATGGGACGCTCTAATACAGACATGGT
GCGAAGAGTCTATTGAGCTAGTTGGTAGTCCTCCGGCCCCTGAATGCGGCTAATCCTAACTG
CGGAGCACACACCCTCAAGCCAGAGGGCAGTGTGTCGTAACGGGCAACTCTGCAGCGGAA
CCGACTACTTTGGGTGTCCGTGTTTCATTTTATTCCTATACTGGCTGCTTATGGTGACAATTG
AGAGATCGTTACCATATAGCTATTGGATTGGCCATCCGGTGACTAATAGAGCTATTATATATCC
CTTTGTTGGGTTTATACCACTTAGCTTGAAAGAGGTTAAAACATTACAATTCATTGTTAAGTT
GAATACAGCAAAACTAGTGCCACC**ATG**TTCGTGTTCCTGGTGCTGCTGCCCCTGGTGAGCA
GCCAGTGCGTGGTTCCTCAGGCTCTCCTGTTTGTACCCCTTCTGGTTTTTCCATTGTGTTTTG
GGAAATTCCCTATTTACACGATACCAGACAAGCTTGGTCCCTGGAGTCCGATTGACATACAT
CACCTCAGCTGCCCAAACAATTTGGTAGTGGAGGACGAAGGATGCACCAACCTGTCAGGG
TTCTCCTACATGGAACTTAAAGTTGGATACATCTTAGCCATAAAAGTGAACGGGTTCACTTG
CACAGGCGTTGTGACGGAGGCTGAAACCTACACTAACTTCGTTGGTTATGTCACAACCACG
TTCAAAAGAAAGCATTTCCGCCCAACACCAGATGCATGTAGAGCCGCGTACAACTGGAAG
ATGGCCGGTGACCCCAGATATGAAGAGTCTCTACACAATCCGTACCCTGACTACCGCTGGCT
TCGAACTGTAAAAACCACCAAGGAGTCTCTCGTTATCATATCTCCAAGTGTGGCAGATTTGG
ACCCATATGACAGATCCCTTCACTCGAGGGTCTTCCCTAGCGGGAAGTGCTCAGGAGTAGC
GGTGTCTTCTACCTACTGCTCCACTAACCACGATTACACCATTTGGATGCCCGAGAATCCGA
GACTAGGGATGTCTTGTGACATTTTTACCAATAGTAGAGGGAAGAGAGCATCCAAAGGGAG
TGAGACTTGCGGCTTTGTAGATGAAAGAGGCCTATATAAGTCTTTAAAAGGAGCATGCAAA
CTCAAGTTATGTGGAGTTCTAGGACTTAGACTTATGGATGGAACATGGGTCTCGATGCAAAC
ATCAAATGAAACCAAATGGTGCCCTCCCGATAAGTTGGTGAACCTGCACGACTTTCGCTCA
GACGAAATTGAGCACCTTGTTGTAGAGGAGTTGGTCAGGAAGAGAGAGGAGTGTCTGGAT
GCACTAGAGTCCATCATGACAACCAAGTCAGTGAGTTTCAGACGTCTCAGTCATTTAAGAA
AACTTGTCCCTGGGTTTGGAAAAGCATATACCATATTCAACAAGACCTTGATGGAAGCCGAT
GCTCACTACAAGTCAGTCAGAACTTGGAATGAGATCCTCCCTTCAAAAGGGTGTTTAAGAG
TTGGGGGGAGGTGTCATCCTCATGTGAACGGGGTGTTTTTCAATGGTATAATATTAGGACCT
GACGGCAATGTCTTAATCCCAGAGATGCAATCATCCCTCCTCCAGCAACATATGGAGTTGTT
GGAATCCTCGGTTATCCCCCTTGTGCACCCCCTGGCAGACCCGTCTACCGTTTTCAAGGACG
GTGACGAGGCTGAGGATTTTGTTGAAGTTCACCTTCCCGATGTGCACAATCAGGTCTCAGG
AGTTGACTTGGGTCTCCCGAACTGGGGGAAGTATGTATTACTGAGTGCAGGGGCCCTGACT
GCCTTGATGTTGATAATTTTCCTGATGACATGTTGTAGAAGAGTCAATCGATCAGAACCTAC
GCAACACAATCTCAGAGGGACAGGGAGGGAGGTGTCAGTCACTCCCCAAAGCGGGAAGA
TCATATCTTCATGGGAATCACACAAGAGTGGGGGTGAGACCAGACTG**TGATAATAG**ACCGG
TAAAAAACAAAAAACAAAACGGCTATTATGCGTTACCGGCGAGACGCTACGGACTTAAATA

ATTGAGCCTTAAAGAAGAAATTCTTTAAGTGGATGCTCTCAAACTCAGGGAAACCTAAATC
TAGTTATAGACAAGGCAATCCTGAGCCAAGCCGAAGTAGTAATTAGTAAGACCAGTGGACA
ATCGACGGATAACAGCATATCTAGTTT-3'

Table S3. The sequences of circRNA-RBD produced via group I intron.

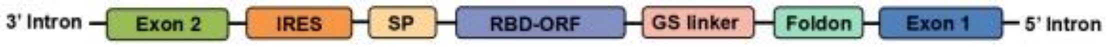

IVT template_circRNA-RBD

5'-
AACAATAGATGACTTACAACTAATCGGAAGGTGCAGAGACTCGACGGGAGCTACCCTAACG
TCAAGACGAGGGTAAAGAGAGAGTCCAATTCTCAAAGCCAATAGGCAGTAGCGAAAGCTG
CAAGAGAATGAAAATCCGTTGACCTTAAACGGTCGTGTGGGTTCAAGTCCCTCCACCCCCA
CGCCGGAAACGCAATAGCCGAAAAACAAAAAACAAAAAAAACAAAAAAAAAACCAAAA
AAACAAAACACATTAAAACAGCCTGTGGGTTGATCCCACCCACAGGCCCATTGGGCGCTAG
CACTCTGGTATCACGGTACCTTTGTGCGCCTGTTTTATACCCCCTCCCCCAACTGTAACTTAG
AAGTAACACACACCGATCAACAGTCAGCGTGGCACACCAGCCACGTTTTGATCAAGCACTT
CTGTTACCCCGGACTGAGTATCAATAGACTGCTCACGCGGTTGAAGGAGAAAGCGTTCGTT
ATCCGGCCAACTACTTCGAAAAACCTAGTAACACCGTGGAAGTTGCAGAGTGTTTCGCTCA
GCACTACCCCAGTGTAGATCAGGTCGATGAGTCACCGCATTCCCCACGGGCGACCGTGGCG
GTGGCTGCGTTGGCGGCCTGCCCATGGGGAAACCCATGGGACGCTCTAATACAGACATGGT
GCGAAGAGTCTATTGAGCTAGTTGGTAGTCCTCCGGCCCCTGAATGCGGCTAATCCTAACTG
CGGAGCACACACCCTCAAGCCAGAGGGCAGTGTGTCGTAACGGGCAACTCTGCAGCGGAA
CCGACTACTTTGGGTGTCCGTGTTTCATTTTATTCCTATACTGGCTGCTTATGGTGACAATTG
AGAGATCGTTACCATATAGCTATTGGATTGGCCATCCGGTGACTAATAGAGCTATTATATATCC
CTTTGTTGGGTTTATACCACTTAGCTTGAAAGAGGTTAAAACATTACAATTCATTGTTAAGTT
GAATACAGCAAAACTAGTGCCACC**ATG**TTCGTGTTCCTGGTGCTGCTGCCCCTGGTGAGCA
GCCAGTGCGTGAGGGTGCAGCCCACCGAGTCCATCGTGAGGTTTCCTAACATCACCAATCT
GTGTCCCTTCGGAGAGGTGTTCAATGCCACAAGGTTCGCCTCTGTGTATGCATGGAACCGC
AAGAGGATTTCCAACTGCGTGGCTGATTACTCAGTGCTGTACAATTCAGCCAGCTTCTCCAC
ATTTAAGTGCTATGGCGTGTCTCCAACTAAGCTGAACGATCTGTGCTTCACAAACGTGTACG
CCGACAGCTTCGTGATCCGCGGCGATGAAGTGAGACAGATCGCCCCAGGCCAGACCGGCA
AGATTGCCGACTACAACTACAAGCTGCCTGATGATTTCACAGGCTGCGTGATTGCCTGGAA
CAGCAACAATCTGGACAGCAAGGTGGGAGGCAATTACAACTACCTGTACCGCCTGTTCAGG
AAGAGCAACCTCAAGCCTTTCGAGAGGGACATCAGCACCGAGATCTACCAGGCTGGTTCTA
CCCCATGTAACGGCGTGGAGGGGTTTAACTGCTACTTCCCCCTGCAGAGTTACGGCTTCCA
GCCTACCAACGGAGTGGGCTACCAGCCATACCGCGTGGTGGTGCTGTCCTTCGAGCTGCTG
CACGCTCCAGCCACCGTGTGTGGCCCTAAGAAATCTACAAACCTGGTGAAGAACAAGTGC
GTGAACTTTGGCGGAGGAGGCAGCGGCGGAGGAGGCAGCGGAAGCGGCTACATCCCAGA
AGCCCCTAGAGACGGACAGGCTTACGTGCGAAAAGACGGCGAGTGGGTGCTGCTGAGCAC
ATTCCTGGGAAGGAGC**TGATAATAG**ACCGGTAAAAAACAAAAAACAAAACGGCTATTATG
CGTTACCGGCGAGACGCTACGGACTTAAATAATTGAGCCTTAAAGAAGAAATTCTTTAAGT
GGATGCTCTCAAACTCAGGGAAACCTAAATCTAGTTATAGACAAGGCAATCCTGAGCCAAG
CCGAAGTAGTAATTAGTAAGACCAGTGGACAATCGACGGATAACAGCATATCTAGTTT-3'
